# A Randomized Controlled Trial on The Beneficial Effects of Training Letter-Speech Sound Integration on Reading Fluency in Children with Dyslexia

**DOI:** 10.1371/journal.pone.0143914

**Published:** 2015-12-02

**Authors:** Gorka Fraga González, Gojko Žarić, Jurgen Tijms, Milene Bonte, Leo Blomert, Maurits W. van der Molen

**Affiliations:** 1 Department of Developmental Psychology, University of Amsterdam, Amsterdam, The Netherlands; 2 Rudolf Berlin Center, Amsterdam, The Netherlands; 3 Department of Cognitive Neuroscience, Faculty of Psychology and Neuroscience, Maastricht University, Maastricht, The Netherlands; 4 Maastricht Brain Imaging Center, Maastricht University, Maastricht, The Netherlands; 5 IWAL Institute, Amsterdam, The Netherlands; 6 Amsterdam Brain and Cognition, University of Amsterdam, Amsterdam, The Netherlands; MRC Institute of Hearing Research, UNITED KINGDOM

## Abstract

A recent account of dyslexia assumes that a failure to develop automated letter-speech sound integration might be responsible for the observed lack of reading fluency. This study uses a pre-test-training-post-test design to evaluate the effects of a training program based on letter-speech sound associations with a special focus on gains in reading fluency. A sample of 44 children with dyslexia and 23 typical readers, aged 8 to 9, was recruited. Children with dyslexia were randomly allocated to either the training program group (n = 23) or a waiting-list control group (n = 21). The training intensively focused on letter-speech sound mapping and consisted of 34 individual sessions of 45 minutes over a five month period. The children with dyslexia showed substantial reading gains for the main word reading and spelling measures after training, improving at a faster rate than typical readers and waiting-list controls. The results are interpreted within the conceptual framework assuming a multisensory integration deficit as the most proximal cause of dysfluent reading in dyslexia.

***Trial Registration***: ISRCTN register ISRCTN12783279

## Introduction

Dyslexia is a specific reading and spelling disability with a neurobiological basis and prevalence estimates between 3% and 10% depending on the study and precise assessment criteria [1,2]. The most characterizing symptom is a persistent failure to develop fluent reading skills [3,4]. These impairments can have severe academic, economic and psychosocial consequences, thus requiring clinical intervention [5].

During the last decades, research focused on the phonological theory of dyslexia. Accordingly, the ability to attend to and manipulate speech sounds, referred to as phonological awareness, is impaired in dyslexic readers, hindering the acquisition of reading skills [4,6]. Nonetheless, concerns can be raised regarding the causal role of phonological awareness in dyslexia [7,8]. Firstly, as concluded in a review of the pertinent literature by Castles & Coltheart (2004) [9], there is still no convincing evidence that phonological awareness precedes and directly influences reading acquisition. The results of a study in which a group of preliterate children was provided with either phonemic awareness training, letter awareness training or a control task, followed by teaching the alphabetic principle and decoding skills are in line with this conclusion [10]. The results of this study revealed that, although phonemic awareness training was successful in itself, it had no effect on the subsequent acquisition of reading skills. Along similar lines, Blomert & Willems (2010) [8], showed that only a small part of preliterate children at risk for dyslexia present phonemic awareness problems in kindergarten, and that 80% of the at-risk children who later develop a reading deficit do not reveal a phonemic awareness problem in kindergarten. Secondly, phonological awareness has been shown to develop as a consequence rather than as a precursor of reading acquisition [7,11–14], but see [15]. Thirdly, a phonological awareness deficit fails to explain why, especially for (semi-) transparent languages, dysfluent reading is the most persistent symptom of dyslexia, and why, even when phonological awareness and visual word decoding skills are adequate, dyslexic reading remains dysfluent [16,17].

A rapidly growing body of research thus focuses on a letter-speech sound binding deficit as the most proximal cause for dyslexia [18–22]. The development of grapheme-phoneme associations is considered essential for the acquisition of fluent reading skills [23,24]. Accordingly, knowledge of these correspondences is used to link spelling of written words to their pronunciation and meaning. This enables sight word learning, that is, automatic and accurate word reading from memory [23]. If the grapheme-phoneme mapping is not correctly automatized, acquiring normal levels of fluency in word reading may require much more time and practice [25]. Moreover, these associations may support the development of phonological awareness for isolated speech sounds during reading acquisition. Additionally, previous studies suggested that temporal processing (unimodal and cross-modal) may contribute to reading deficits in dyslexia, emphasizing speed of integration as a critical factor [26,27].

Neuroimaging studies suggested that the network for multimodal processing in left temporo-parietal brain regions is involved in letter-sound integration [17,28–31]. It has been suggested that this network develops first during reading acquisition and then supports the subsequent specialization of occipito-temporal areas for visual word recognition [17,32,33]. Dysregulation in the temporo-parietal and occipito-temporal networks for reading have been found in dyslexics [4,30,34–38]. Interestingly, deviant processing of letters and speech sounds in the multisensory temporo-parietal brain areas has been reported in dyslexic children even if they attained adequate knowledge about letter-speech sound correspondences [30,39]. Additionally, activation in these brain areas correlates to the speed of performance in letter-speech sound matching tasks [30]. In yet another study, reduced activation in integration areas was observed to be directly associated with a deficit in the auditory processing of speech sounds, which in turn predicted performance on phonological tasks [40]. Similarly, brain studies examining preliterate children at risk of dyslexia suggested that neural deficits in auditory processing in temporal and parietal areas could be used as early predictors of reading impairments [15,41–42].

Collectively, the findings reviewed above support the notion that reduced letter-sound integration qualifies as the proximal cause of the reading failure in dyslexics. Comparable results have been reported in a cognitive study by Aravena et al. (2013) [43], who developed a task for letter-speech sound learning in an artificial script. The results of this study showed that children with dyslexia attained levels of letter-speech sound knowledge comparable to those of their normal reading peers, but their level of letter-speech sound mapping fluency was significantly lower than that of normal reading children. These results indicate that letter-speech sound knowledge is not sufficient to develop automated letter-speech sound integration, and suggest that children with dyslexia have a specific deficit in this speeded integration [43].

Current interventions for dyslexia show that reasonable levels of accuracy in reading may be attainable [44–47]. However, they still do not provide an effective remediation for the lack of reading fluency [3,48–53]. A typical example is the study of Torgesen and colleagues, in which dyslexic children received 67.5 hours of treatment on phonemic awareness and phonemic decoding skills [54]. Results revealed large effects on reading accuracy, children’s average scores on accuracy were within the average range after treatment. In contrast, dyslexics’ standard scores in reading fluency were virtually unchanged, 96% to 100% of the children were still below the average range on after treatment [54]. Importantly, the training specific effects on addressing the ‘fluency barrier’ in dyslexia are still unclear (see review in [55]). As concluded by Elliott & Grigorenko (2014) [56], training of alphabetic principle and decoding skills, despite long-lasting assumptions to the contrary, does not appear to lead to improved reading fluency (p. 171).

Inspired by the multisensory integration deficit account (e.g., [17]), assuming a failure to develop automatic letter-speech sound integration in dyslexia, the present study will examine a cognitive training focusing on the development of automated letter-speech sound integration. The current training provides for systematic practice on regular and irregular letter-speech sound mappings at increasing levels of complexity. Importantly, the attainment of these correspondences is facilitated by intensive exposure to ensure the automation of letter-speech sound mapping and, thus, reading fluency. Furthermore, we used a randomized-controlled trial (RCT) design, including waiting-list dyslexic readers besides age-matched typical readers, and a wide range of outcome measures, for both accuracy and speed, including word reading, spelling and letter-speech sound mapping. This should allow for a detailed assessment of training benefits.

The present evaluation will consist of the following steps. First, we will perform a baseline analysis on test scores to obtain a complete assessment of reading deficits in the dyslexic groups vis-a-vis the typical readers. Subsequently, we will compare reading gains in trained vs. untrained dyslexics in terms of test scores, while accounting for potential group differences in initial performance. Secondly, we will identify latent factors to assess the relation between outcome measures. The latent factors emerging from the principal component analysis (PCA) will be used in the subsequent analyses to facilitate the interpretation of potential effects of training on reading fluency. Thirdly, we will assess baseline differences and training effects between the dyslexic groups in terms of factor scores. This analysis will be followed by a mixed-model analysis to assess between-groups differences relative to the typical readers, in the rate of change on reading fluency during the intervention period. Finally, a correlational analysis will be performed to examine the relation between initial letter-speech sound mapping skills and the development of reading fluency.

In brief, the overall objective of the current study is to broaden our insights in how to remedy reading fluency problems in children with dyslexia. We aim to contribute to current research on remediation programs on dyslexia by providing a detailed window on the relation between training letter-speech sound mappings and reading fluency, using a large number of outcome measures, a strictly controlled and systematized training procedure, and a RCT design.

## Methods

The study was an open randomized controlled trial comparing an intervention addressing letter-speech sound integration to a waiting list control group (allocation ratio 1:1). The approval for the research was obtained from the local ethical committee of the Developmental Psychology department of the University of Amsterdam. All parents or caretakers signed informed consent before the children participated in this study. The protocol for this trial and the supporting CONSORT checklist are available as supporting information; see S1 Checklist and S1 Protocol. This study is registered as ISRCTN12783279 (www.isrctn.com). This trial was registered retrospectively due to the fact that it was not a mandatory requirement to have it registered by our approving Ethical Committee board. The authors confirm that all ongoing and related trials for this intervention are registered.

### Participants

**Fig 1:**
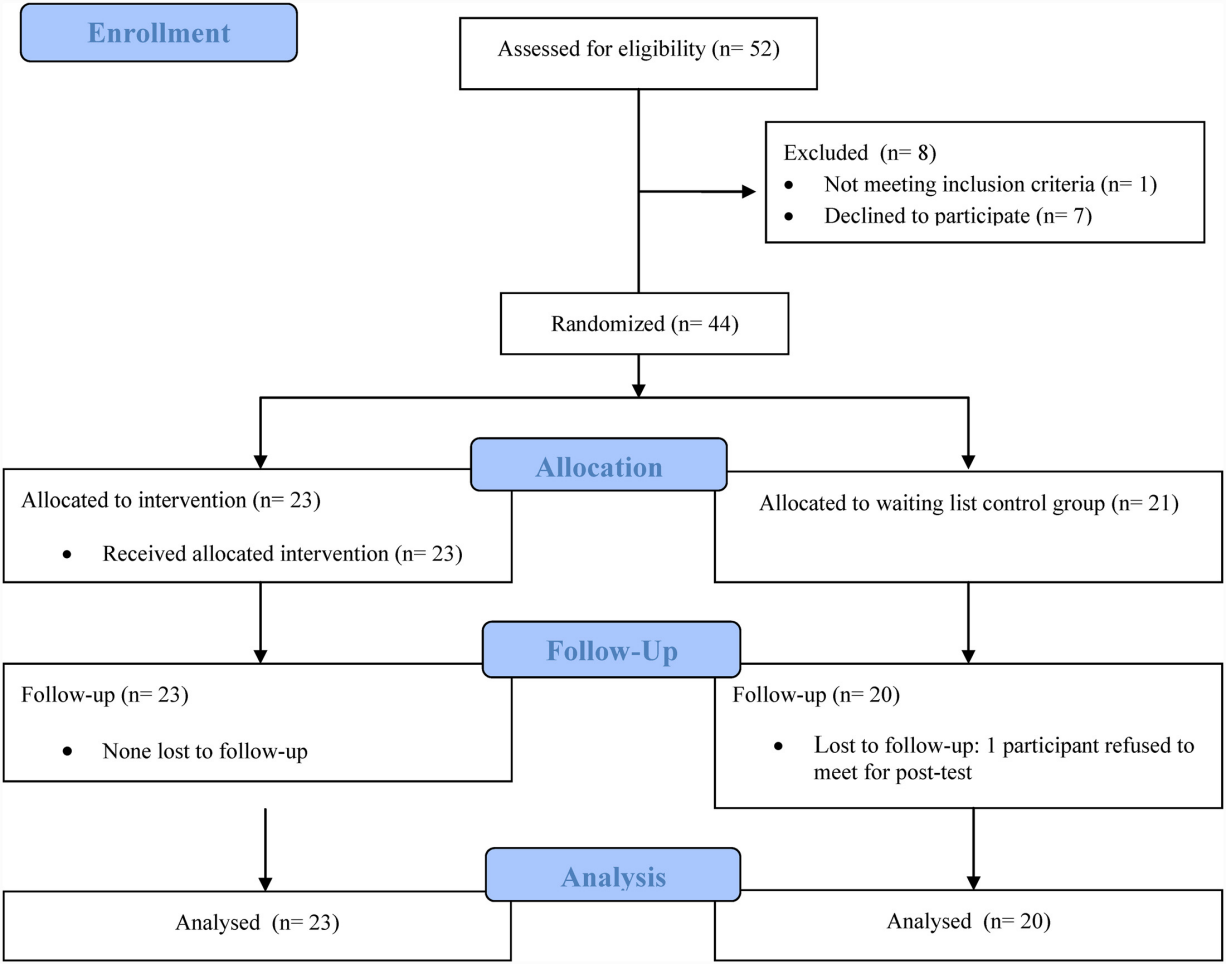
Participant flow diagram.

The inclusion period for the trial was from October 2011 to December 2011. The flow of participants in the study is presented in Fig 1. Third-grade children with the diagnosis of dyslexia (N = 44; 8.86 ± 0.43 years old, 24 boys and 20 girls) were recruited from a nation-wide center for dyslexia in the Netherlands. To be eligible the children had to have a percentile score of 10 or lower on standard reading measures, and to be referred to the center because of persistent and specific reading problems. They were randomly allocated to either the training program group (N = 23; 8.94 ± 0.44 years old, 11 boys and 12 girls) or to a waiting-list control group (N = 21; 8.77 ± 0.41 years old, 13 boys and 8 girls). Participants allocated to the waiting-list control condition received the intervention program after the waiting period had elapsed. Participants were randomized using a computerized random number generator by a staff member not involved in training or testing. Simple randomization was used with no restrictions (e.g., blocking or stratification). A group consisting of 23 third-grade, typical readers (8.67 ± 0.34 years old, 9 boys and 14 girls) was recruited from several primary schools attended by children with the same sociodemographical background as the dyslexic group (see Table 1 for group characteristics). To be eligible, they had to have no history of reading difficulties, and a percentile score of 25 or higher on standard reading tests (see below). One child of the waiting-list control group dropped out, resulting in a sample of 20 children for the post-test measures. The post-test scores for the 3DM word-reading task were discarded for one child of the training group who obtained extremely low accuracy scores (below 3 x Inter Quartile Range). Additional missing values in some of the outcome measures were due to computer failure (see footnotes in the corresponding tables).

**Table 1:** Descriptive statistics showing demographics and IQ.

	Typical Readers	Dyslexics Control	Dyslexics Training	group differences	
	*M (SD)*	*M (SD)*	*M (SD)*	*F*	*p-value*
*N*	23	21	23		
Sex ratio (m:f)	9:14	13:8	11:12		
Age	8.68 (0.34)	8.82 (0.33)	8.94 (0.44)	2.76	.071
RAVEN—IQ test [C]^a^	7.19 (1.48)	6.80 (1.50)	7.48 (1.35)	1.24	.297

^a^ C scores (M = 5, SD = 2).

All participants were native Dutch speakers, received two and a half years of formal reading instruction in primary education. The RAVEN Coloured Progressive Matrices (RAVEN CPM) was used as a control non-verbal measurement of IQ to obtain an estimate of fluid intelligence [57]. Additionally, the Child Behavior Checklist (CBCL) was completed by the parents to exclude any additional behavioral problems [58]. Children with below average IQ (IQ < 85 on a non-verbal IQ-test), uncorrected sight problems, hearing loss, diagnosis of ADHD or other neurological or cognitive impairments were excluded. Both the clinical center and the schools participating were located in the Amsterdam area.

Sample size calculation (Power & Precision V4 software; [59]) indicated that a sample size of n ≥ 20 per intervention condition would be required for a power of at least 0.80 to detect an intervention effect of medium to large effect size for gains in reading fluency, for an ANCOVA with 1 factor (intervention condition) and 1 covariate (pre-test level, *R* ^*2*^ = .37, based on previous intervention studies), and α = 0.05.

### Outcome measures

A series of tests was used to assess the reading skills of the participants. In accordance with our main objective, we considered reading fluency scores as our primary outcome measure, and the other scores as secondary outcome measures. The 3DM battery of tests (test reliability and normative sample information available in Differential Diagnosis; 3DM [60]) contains word reading, phonological awareness, naming speed and letter-speech sound association tasks. This battery is administered individually using a computer and a specialized response-box records reaction time with millisecond accuracy. The scores of the following 3DM subtests have been used in the present study.

#### Word reading task

This task includes three different subtasks containing high-frequency words, low-frequency words and pseudowords. The mean frequencies of the high-frequency words are between 790 and 45810 and for the low-frequency words they range between 6 and 342 (CELEX‐database;[61]). There are 75 words for each level (5 screens with 15 items each). The difficulty of each level increases systematically from monosyllabic words without consonant clusters to 3 or 4 syllabic words with consonant clusters at the fifth level. The participants are asked to read accurately as many words as possible. When they finish reading one screen the experimenter presses a button to continue until the time limit of 30 seconds per subtask is reached. The number of words read correctly within 30 seconds determines the reading fluency score per subtask (r = .91-.93 for the subtasks, and r = .95 for total task, test-retest). The proportion of correctly read words within the time limit accounts for the reading accuracy scores (r = .73, test-retest).

#### Letter-speech sound (LSS) association tasks

Two tasks were used to measure accuracy and automation of letter‐speech sound (LSS) mapping; LSS identification and LSS discrimination. LSS identification requires a child to match a speech sound to one of four presented letter (combinations) by pressing the corresponding button (e.g. /b/ and ‘b’ ‘d’ ‘t’ ‘p’). LSS discrimination asks a child to judge whether a speech sound and letter are congruent or incongruent (e.g. /ui/ and ‘oe’). Accuracy (% correct) as well as response time (sec/item) is measured (LSS identification: r = .72 for accuracy and r = .90 for response time; LSS discrimination: r = .82 for accuracy and r = .96 for response time, internal consistency).

#### Computerized spelling

A word is presented aurally (over headphones) as well as visually (at the computer screen). In the visually presented word, a letter (combination) is missing and the child is instructed to choose the missing part out of four visually presented options by pressing the corresponding button (e.g. auditory stimulus /boom/ (tree), visual stimulus ‘b__m’, options ‘oo’ ‘o’ ‘a’ ‘aa’). Words are spelled either phonetically (18 items) or contain Dutch spelling rules (36 words). Word frequencies are varied systematically. Accuracy (% correct) as well as response time (sec/item) is measured (r = .80 for accuracy and r = .94 for response time, internal consistency).

#### Rapid naming task

The rapid naming (RAN) task consisted of three subtasks: letters, digits and objects. Each subtask contains 5 items repeated six times, distributed in two screens of 15 items. Participants are instructed to name the items as fast and accurate as possible. The score per subtask was determined by taking the mean response time of the two screens (r = .80 for letters, r = .83 for digits, and r = .71 for objects, split-half reliability).

#### Phonological awareness (PA)

An estimate of phonological awareness is obtained by using a phoneme deletion task presenting 23 pseudowords with a CVC or CCVCC structure. The participant must omit a consonant that is either at the beginning or at the end of a word or within a consonant cluster as fast as possible. The score is determined by the percentage of correct responses. (r = .85, internal consistency).

In addition to the 3DM battery the following tests were used.

#### Word reading fluency

The Dutch version of the *One-minute test* (Een-Minuut-Test; [62]), was used to provide an additional estimate of word reading skills. It is a time-limited test consisting of a list of 116 unrelated words of increasing difficulty. The number of correctly read words within 1 minute serves as reading fluency score (r = .90, test-retest).

#### Text reading fluency

The text-reading fluency test consists of a coherent text of increasing difficulty. The child is asked to read the text out loud within one minute (Schoolvaardigheidstoets Technisch Lezen; [63]. Again, the number of correctly read words within 1 minute serves as reading fluency score (r = .88, test-retest).

### Procedure

The study used a pre-test-training-post-test design. Pre-test (period: December 2011 to Januari 2012) and post-test (period June 2012 to July 2012) were administered at either the clinical center for the dyslexic children or at school for the normal readers during a session of approximately one hour. Children are tested individually in a silent room.

The training-program group received an average of 33.65 ± 0.83 sessions while the other two groups received no training. The average number of weeks between pre- and post-test measurements was 22.92 ± 3.51 across the three groups; 20.17 ± 1.56 for typical readers, 25.70 ± 3.33 for the training-program group and 23.26 ± 3.08 for the waiting-list control group. The number of weeks between tests differed between the three groups, *F* (2, 63) = 22.07, *p* = < .001, *η* ^*2*^ = 0.41. Post-hoc comparisons revealed the differences between the two dyslexic groups in the number of weeks was statistically significant, *p* = .015. Thus, comparisons between groups will take into account the difference in the number of weeks elapsing between pre- and post-test.

### Training

Dyslexic children followed an intensive tutor and computer-assisted training program. The program was provided by well-instructed junior psychologists, on a one-to-one basis during 45-min sessions. The training frequency was two sessions per week.

The training is constructed in accordance with general skill acquisition paradigms [64,65], which basically implies that each (letter-speech sound) element is taught explicitly at first and consequently repeated intensively in order to obtain a transition from accurate, controlled to associative, automatic processing. Accordingly, a previous study showed that massive exposure to letter-speech sound correspondences is substantially more effective in automatizing letter-speech sound integration when it is preceded by explicit teaching of these correspondences than when it is presented on its own [43]. Sessions consist therefore in an instruction part and a practice part. In the instruction part the letter-speech sound correspondences are explicitly taught aiming at accurate mastery of the learned associations. During the practice part, the computer training provides a high exposure to the specific letter speech sound associations that were taught during the instruction part, to stimulate the automatic integration of letters and speech sounds.

The training started with the tutor explaining consistent letter-speech sound correspondences. First, the standard letter—speech sound correspondences are being trained and, subsequently, the irregular letter—speech sound mappings. To do so, a reconfigured touchscreen was used that consists of buttons for each Dutch speech sound (see S1 Fig for an illustration of the touchscreen buttons). Each button shows the standard letter or letter-cluster of the corresponding speech sound. In addition, the touchscreen includes several icons to indicate the type of phoneme (e.g., ‘long vowel’), syllable icons (e.g., ‘stressed syllable’) and rule icons to perform operation (e.g., delete a selected grapheme; see kernel algorithm below). During instruction, the tutor asks the child to pronounce the corresponding speech sound, which is presented not only in isolation but also within the context of a (visual) word. Subsequently, the child is asked to identify the item both orally and by pressing the corresponding buttons in the touch screen. When the child presses a button the computer produces the corresponding speech sound (by a natural voice). This is done to ensure that attention is directed to the matching of letters and speech sounds. Throughout the session, the tutor corrects the child if the response would be wrong. Similarly, the computer screen provides performance feedback following erroneous button presses. The letter-speech sound couplings are taught step-by-step, e.g., first the short vowels, then long vowels, and later on diphthongs.

Dutch orthography is considered to be of intermediate complexity (e.g., [66]), which implies that the one-to-one mapping between letters and speech sounds can be broken. To learn these inconsistent correspondences, phonological-orthographic mapping operations are introduced during the second part of the program. These operations follow a uniform inferential algorithm that constitutes the kernel of the present training, i.e.:

IF p/#∈PithenO(p) →g∈G.

*When the terminal phonic element p of a syllable belongs to the i* ^*th*^ *category of phonetic elements Pi then the result of an operator O on p will be mapped onto a graphic element g that need not be the standard mapping*.

The basic principles of the Dutch written language can be structured within a learning system incorporating five types of operations as a consequence of five types of terminal phonic elements; long vowels, short vowels, unvoiced consonants, sonic vowels and unstressed morphemes. For example, in Dutch, voiced consonants (/d/ and /b/) lose the voice property at the terminal position, which is not reflected in their orthographic representation. Consequently, the algorithm prescribes: if the last speech sound in a syllable is an unvoiced consonant then extends the word (operation) and if this results in a voiced consonant the voiced consonant graph should be written (e.g., paard [IPA: part]–paarden [pardən] (English: horse—horses)), otherwise the standard consonant (e.g., kat [kɑt]–katten [kɑtən] (cat—cats)). All these rules and elements are incorporated in the touchscreen (see S1 Fig). Thus, the essential terms in the algorithm have an explicit and exhaustive description in the program with regards to the set of speech sounds, the categories of speech sounds, the corresponding orthographic elements, and the mapping operations. Consequently, the focus of attention remains continuously on the integration of letters and speech sounds.

**Fig 2:**
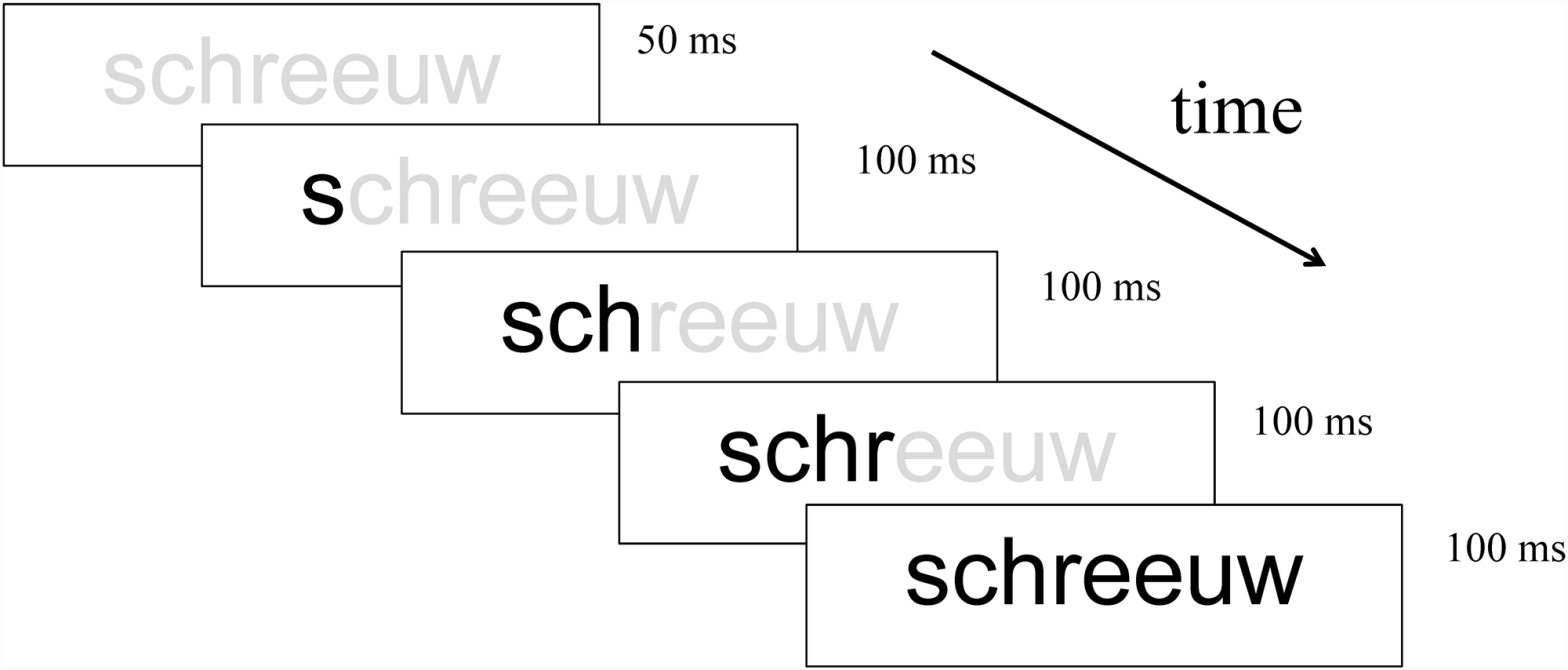
Example of a practice item. The presentation of the word schreeuw [sxreːu] (English: shout) under time-demanding conditions. The visual presentation is sound by sound: s[s] _ ch[x] _ r[r] _ eeuw[eːu]. (IPA symbols in brackets). The participant must pronounce the corresponding (visually presented) sounds and in the end the whole word.

Along with the learning of both consistent and inconsistent letter-speech sound mappings, the computer training provides a high exposure to letter-speech sound mappings at increasing levels of complexity. A typical example of an exercise during practice refers to the projection of individual words, speech sound by speech sound, on the computer screen under (progressive) time demands (see Fig 2). The child is asked to pronounce the word sound by sound (and in the end the whole word), guided by the time-constraints of the graphemic presentation rate. During presentation, the whole word is projected faintly on the screen to allow anticipation (cf., [67]). During a practice session, specific letter-speech sound mappings or clusters of mappings (e.g., all long vowels) are presented, matching those addressed in the preceding instruction part (but in a different body of words from those used during instruction). Practice is adjusted to the individual rate of acquisition by adapting time-constraints to the level of the child’s performance. When at least 80% of the items are correctly executed the participant moves to the next step of the training.

The current training is an adaptation of an existing intervention program. The adaptation consists of an exclusive focus on letter-speech sound mapping integration. An extensive description, including a more detailed illustration of the tutor-participant interaction during sessions, can be found in Tijms et al. (2007) [44].

### Statistical analysis

For the AN(C)OVAs (see below), standardized scores were used instead of raw scores, in order to assess the child’s position within the distribution of a normative sample. For the latent factor analysis, factor scores were obtained from raw scores. In addition, due to reduced variance, no reliable norm scores were available for the accuracy measures of the three subtasks of the 3DM word reading; thus raw scores were used for these measures. The evaluation of potential training effects comprised the following sequence of steps.

First, one-way ANOVAs were performed to examine baseline differences. The outcome of this analysis should provide a first impression of group differences before evaluating training effects. Planned comparisons were then performed between typical readers and the dyslexic groups, and between the two dyslexic groups at pre-test. Then, in order to test potential training effects, ANCOVAs were performed comparing the two groups of dyslexics, using post-test scores as dependent variables and the corresponding pre-test scores as covariate (one-tailed *p* values are reported). This approach was selected because of its higher statistical power in randomized studies relative to other methods of analysis [68]. In order to account for potential effects of between group differences in pre- to post-test interval, an additional control analysis was performed including the number of weeks between tests as a covariate. The pattern of results did not differ between the two analyses, thus we will report only the results of the ANCOVA with pre-test scores as a covariate.

Subsequently, Principal Component Analysis (PCA), with varimax rotation, was performed in order to reduce the number of outcome variables by taking into account the relations between measures. This should facilitate the interpretation of the potential training effects [69]. The analysis was performed including pre-test data from the entire sample consisting of the main 3DM fluency and accuracy scores associated with word reading (high frequency, low frequency and pseudowords), spelling and LSS identification and discrimination. Only the 3DM scores were used as they are part of the assessment battery that is most used in diagnosis of dyslexia in the Netherlands and constitute the primary measures in the current study. Factors were extracted using the eigenvalue-one procedure. We obtained factor scores (with mean zero) weighted by regression coefficients obtained by multiplying the inverse of the variables correlation matrix by the matrix of factor loadings. The same procedure was applied to post-test data to obtain factor scores used in the subsequent analyses. Baseline differences between groups and potential training effects were examined by submitting the factor scores to, respectively, one-way ANOVA and ANCOVA.

Additionally, we used a mixed model to evaluate rate of change on the extracted factor scores between pre and post-test between the three groups. This allows for examining the relationship between covariate and dependent variables across groups [70]. In addition, it is suited for longitudinal data analysis and can handle missing values (see participants section for details about attrition and missing values). The present analysis used a random intercept model including three groups and accounting for significant baseline differences between typical readers and dyslexics. The fixed part of the model included the factor group, time (pre-post-test) and their interaction. The factor scores were used as dependent variable. The analysis focused on the fixed effects estimates for the interaction of each group with the factor time. The group of typical readers was used as a reference, as they are expected to having attained high levels of reading fluency and therefore to exhibit the lowest improvement rate. The estimates for dyslexics training and dyslexics control were then compared to those of the typical readers group.

Finally, we examined the relationship between letter-speech sound mapping skills and reading improvement. For this purpose, partial correlations were performed between the pre-test letter-speech sound fluency factor score and the post-test word reading fluency scores (controlled for pre-test differences).

## Results

### AN(C)OVAs

#### Baseline

The results of the ANOVAs performed on the pre-test standard scores in reading accuracy and speed measures are displayed in Table 2. Levene’s test was significant for the accuracy measures of the 3DM word reading tasks (high frequency words, *F* (2, 64) = 11.42, *p* = .000, low frequency words, *F* (2, 64) = 11.67, *p* = .000, pseudowords, *F* (2, 64) = 4.71, *p* = .012 and overall score, *F* (2, 64) = 3.57, *p* = .034), as well as for the accuracy scores associated with letter-speech sound identification; *F* (2, 64) = 3.74, *p* = .029. So in the first contrast (dyslexics vs. typical readers) the degrees of freedom for these measures were adjusted from 64 to 43.01, 49.21, 62.96, 56.41 and 60.90, respectively. In the second contrast (dyslexics training vs. waiting-list group) the adjusted degrees of freedom were 38.01, 41.73, 41.03, 40.15 and 41.37. The table shows a deficit in dyslexics that is mainly manifested by large differences in the reading fluency measures. Overall, the three groups attained reasonably high levels of accuracy, with the exception of the spelling task where dyslexics performed on average below the 10^th^ percentile. For the majority of the tests, the two dyslexic groups showed significantly lower levels than those of the typical readers. With regard to the 3DM letter-speech sound measures, the results are somewhat more diffuse. The scores of the two dyslexic groups were significantly below those of the typical readers for most tasks with the exception of the fluency score associated with letter-speech sound discrimination (*p* = .347) and the accuracy scores associated with letter-speech sound identification (*p* = .100). In addition, the results showed differences between the two dyslexic groups in letter-speech sound fluency scores, but not in the accuracy scores.

**Table 2:** Descriptive statistics showing reading accuracy and fluency scores at pre-test.

	T		DC		DT		Contrasts					
	*N* = 23		*N* = 21		*N* = 23		DT & DC: T			DC:DT		
	*M*	*SD*	*M*	*SD*	*M*	*SD*	*t*	*p*-value	*Cohen's d*	*t*	*p*-value	*Cohen's d*
3DM Word reading—*accuracy* ^a^												
High Frequency^d^	99.15	1.10	94.34	4.51	92.62	6.98	6.24	.000	1.34	0.98	.498	0.29
Low Frequency^d^	97.40	3.15	86.41	12.86	84.59	15.31	5.35	.000	1.17	0.43	.806	0.13
Pseudowords^d^	87.99	9.13	69.88	17.14	73.03	16.13	5.24	.000	1.23	-0.62	.718	-0.19
Total [T]^b^ ^,^ ^d^	50.13	8.66	31.62	9.92	33.96	13.58	6.84	.000	1.66	-0.66	.718	-0.20
3DM Word reading—*fluency* [T]												
High Frequency	52.83	7.11	32.00	5.88	31.04	5.35	13.42	.000	3.34	0.51	.752	0.17
Low Frequency	54.09	8.54	31.43	5.64	32.09	6.30	12.44	.000	3.04	-0.31	.857	-0.11
Pseudowords	52.48	9.13	29.81	6.36	31.13	5.63	11.84	.000	2.85	-0.61	.718	-0.22
Total	53.52	8.82	30.76	4.62	30.52	5.41	13.48	.000	3.19	0.12	.934	0.05
One-Minute Test -*fluency* [SS]^c^	11.35	2.67	3.76	2.00	3.65	1.87	13.40	.000	3.34	0.16	.934	0.06
Text Reading—*fluency*[T]^1^	54.04	7.82	33.11	5.82	33.83	6.06	11.86	.000	2.97	-0.35	.851	-0.12
3DM Spelling–*accuracy*[T]	50.43	8.54	35.57	6.08	36.87	8.31	7.11	.000	1.79	-0.55	.741	-0.18
3DM Spelling—*fluency*[T]	54.30	8.25	36.05	6.36	40.78	8.46	7.93	.000	1.96	-2.01	.092	-0.63
LSS identification—*accuracy*[T]^d^	47.52	7.50	40.71	10.35	45.13	12.87	1.96	.100	0.45	-1.26	.347	-0.38
LSS discrimination—*accuracy*[T]^2^	50.74	9.05	41.55	8.34	46.52	9.47	2.88	.011	0.72	-1.81	.131	-0.56
LSS identification—*fluency* [T]	52.57	6.66	41.48	7.71	46.17	7.35	4.69	.000	1.19	-2.15	.070	-0.62
LSS discrimination—*fluency*[T]^2^	50.43	7.70	44.45	9.12	50.96	8.48	1.25	.347	0.29	-2.53	.029	-0.74
3DM phoneme deletion—*accuracy*[T]^3^	53.09	7.57	40.38	7.26	37.90	0.12	6.71	.000	1.76	1.01	.498	0.31
3DM Naming speed scores[T]^3^												
Letters	49.65	7.59	37.48	7.85	37.22	7.75	6.19	.000	1.58	-0.11	.934	0.03
numbers	50.43	11.32	38.81	8.35	36.61	8.77	5.15	.000	1.28	0.76	.653	0.26
objects	50.70	7.07	41.43	11.77	41.56	9.42	3.75	.000	1.03	0.05	.962	-0.01
Total	49.96	8.08	36.05	8.78	35.56	9.04	6.36	.000	1.66	0.18	.934	0.05

T = typical readers; DT = dyslexics-training; DC = dyslexics control; LSS = Letter-speech sound.

^a^ Raw scores.

^b^ T scores (M = 50, SD = 10).

^c^ SS scores (M = 10, SD = 3).

^d^ Statistics for equal variances not assumed (*p <* .*05* in Levene's test).

^1^ Data missing for 2 participants: N_DC_ = 19.

^2^ Data missing for 1 participant: N_DC_ = 20.

^3^Data missing for 2 participants N_DT_ = 21.

False Discovery Rate (FDR) correction for multiple comparisons was applied to the *p* values.

#### Training

The two dyslexic groups were compared with regard to their post-test scores, including pre-test scores as covariate. The results are displayed in Table 3. Importantly, the table shows that the training-group dyslexics outperformed waiting-group dyslexics after the letter-speech sound training program. The most substantial differences were present in reading fluency, as expressed by the large effect size of the gains in total reading fluency. This gain in reading fluency holds for high frequency, low frequency and pseudowords. Obviously, training effects were less pronounced for reading accuracy. This absence of substantial effects was to be expected in view of the relatively high accuracy scores prior to training. The training-group dyslexics outperformed waiting-list controls in total reading accuracy score but significance was absent for the three word-type subtests. Finally, with regard to the letter-speech sound mapping tasks, the training-group dyslexics showed significant gains in spelling accuracy, spelling fluency, and fluency associated with letter-speech sound identification relative to the control-group dyslexics.

**Table 3:** ANCOVA comparing dyslexics training and control group in post-test scores with pre-test as covariate.

	Dyslexics control		Dyslexics training		ANCOVA (pre-test as covariate)		
	N = 21		N = 23				
	*M*	*SD*	*M*	*SD*	*F*	*p-value* (one-sided)	*η* ^2^
3DM Word reading—*accuracy* ^a^ ^,^ ^1^ ^,^ ^2^							
High Frequency	94.68	4.02	97.55	2.91	9.21	.006	0.19
Low Frequency	91.27	8.69	93.42	7.90	1.21	.161	0.03
Pseudowords	71.76	18.35	81.82	14.23	3.27	.062	0.08
Total [T]^b^ ^,^	32.85	10.23	41.32	12.81	4.49	.040	0.10
3DM Word reading—*fluency* [T]^1^ ^,^ ^2^							
High Frequency	32.85	5.66	37.36	6.56	14.28	.001	0.27
Low Frequency	30.25	5.02	36.55	6.13	25.39	.001	0.40
Pseudowords	28.25	5.36	33.91	6.63	8.46	.008	0.18
Total	29.60	4.15	35.36	6.40	30.30	.001	0.44
One-Minute Test -*fluency* [SS]^c^ ^,^ ^1^	3.20	1.94	4.09	2.41	3.91	.080	0.09
Text Reading—*fluency*[T]^3^	33.10	5.51	35.04	6.68	1.92	.116	0.05
3DM Spelling–*accuracy*[T]^1^	37.25	6.16	44.91	10.08	10.48	.004	0.21
3DM Spelling—*fluency*[T]^1^	35.20	8.45	44.39	10.23	5.15	.035	0.11
LSS identificacion—*accuracy*[T]^4^	44.00	10.26	46.22	7.84	0.09	.384	0.00
LSS discrimination—*accuracy*[T]^3^	43.58	8.14	47.78	9.50	1.20	.161	0.03
LSS identificacion—*fluency* [T]^4^	40.74	11.28	48.83	9.50	3.86	.052	0.09
LSS discrimination—*fluency* ^3^	48.21	10.15	54.22	9.82	0.61	.236	0.02

LSS = Letter-speech sound.

^a^ Raw scores.

^b^ T scores (M = 50, SD = 10).

^c^ SS scores (M = 10, SD = 3).

^1^ Data missing for 1 participant: N_DC_ = 20.

^2^ Data missing for 1 participant: N_DT_ = 22.

^3^ Data missing for 3 participants: N_DC_ = 18.

^4^ Data missing for 2 participants: N_DC_ = 19.

False Discovery Rate (FDR) correction for multiple comparisons was applied to the *p* values.

### Latent factors analysis

The PCA with varimax rotation was conducted on speed and accuracy measures associated with word reading (high frequency, low frequency and pseudowords), spelling, and letter-speech sound identification and discrimination. Three factors were extracted using the eigenvalue-one procedure. The factors (Eigenvalues = 7.34, 2.18 and 1.28) accounted for, respectively, 38.33%, 21.26% and 17.58% of the variance. The factor loadings are shown in Table 4. The scores that loaded highly on Factor 1 were related to word reading speed and accuracy measures, thus this factor was labelled ‘word reading’. The scores that loaded highly on Factor 2 were related to spelling fluency and fluency associated with letter-speech sound association (identification and discrimination). Thus, this factor was labeled ‘mapping fluency’. Finally, scores related the accuracy of identification and discrimination and spelling accuracy loaded highly on the Factor 3, which was then labeled ‘mapping accuracy’.

**Table 4:** Varimax Rotated Factor Loadings.

Measure	Factor 1	Factor 2	Factor 3
	Word reading	mapping fluency	mapping accuracy
Word reading—accuracy—Total	**.96**	.01	.15
Word reading—accuracy—Low Frequency	**.92**	.05	.06
Word reading—accuracy—Pseudowords	**.78**	.07	.27
Word reading—accuracy—High Frequency	**.76**	-.19	.04
Word reading—fluency- Low Frequency	**.73**	-.44	.43
Word reading—fluency- Total	**.71**	-.47	.45
Word reading—fluency—Pseudowords	**.69**	-.41	.45
Word reading—fluency—High Frequency	**.65**	-.52	.44
LSS identificacion—fluency	-.12	**.87**	-.09
Spelling—fluency	-.33	**.86**	-.01
LSS discrimination—fluency	.18	**.74**	.09
LSS identificacion—accuracy	.02	-.01	**.79**
LSS discrimination—accuracy	.22	.09	**.75**
Spelling—accuracy	.51	-.19	**.61**

LSS = Letter-speech sound. Factor loadings >. 60 are in boldface. All pre-test raw scores from 3DM test. Factor 1 accounted for 38.33% of the variance, Factor 2 accounted for 21.26% of the variance and Factor 3 accounted for 17.58% of the variance, after rotation of Sums of Squared Loadings. Note that LSS fluency scores refer to reaction times while word reading fluency scores refer to number of words per minute.

Group performance in terms of the latent factors loadings is presented in Table 5. The results are clear-cut. That is, the results for all three factors are similar for the two dyslexic groups; both groups differ significantly from the typical readers. The training effects are presented in Table 6. It can be seen that the training-group dyslexics improved significantly with regard to the word reading factor relative to the waiting-list group who showed little if any improvement. The dyslexic groups did not differ with regard to the two mapping factors.

**Table 5:** Descriptive statistics showing baseline differences in factor scores.

	T		DC^1^		DT		Contrasts					
	*N* = 23		*N* = 21		*N* = 23		DT & DC: T			DC:DT		
	*M*	*SD*	*M*	*SD*	*M*	*SD*	*t*	*p*-value	*Cohen's d*	*t*	*p*-value	*Cohen's d*
Word reading ^a^	.77	0.39	-.26	0.85	-.54	1.09	-6.88	.000	1.57	0.94	.354	0.29
Mapping fluency ^a^	-.73	0.61	.61	0.95	.20	0.93	-5.92	.000	-1.4	1.42	.162	0.44
Mapping accuracy	.52	0.69	-.44	0.85	-.14	1.17	-3.34	.001	0.91	-1.06	.293	-0.33

^a^ Statistics for unequal variances (*p <* .*05* in Levene's test).

^1^ Data missing for 1 participant: N_DC_ = 20.

Abbreviations: T, typical readers; DT, dyslexics-training; DC, dyslexics control.

**Table 6:** ANCOVA comparing dyslexics training and control group in post-test factor scores with pre-test as covariate.

	DC		DT		ANCOVA (pre-test as covariate)		
	N = 21^a^		N = 23 ^b^				
	*M*	*SD*	*M*	*SD*	*F*	*p-value* (one-sided)	η^2^
Word reading	-.58	1.26	-.16	0.94	3.42	.037	0.09
Mapping fluency	-.72	1.19	-.18	0.78	1.27	.133	0.03
Mapping accuracy	-.48	0.97	-.07	1.09	0.57	.228	0.02

DT = dyslexics-training; DC = dyslexics control.

^a^ Valid cases for DC = 17.

^b^ Valid cases for DT = 22.

### Mixed model analysis

**Fig 3:**
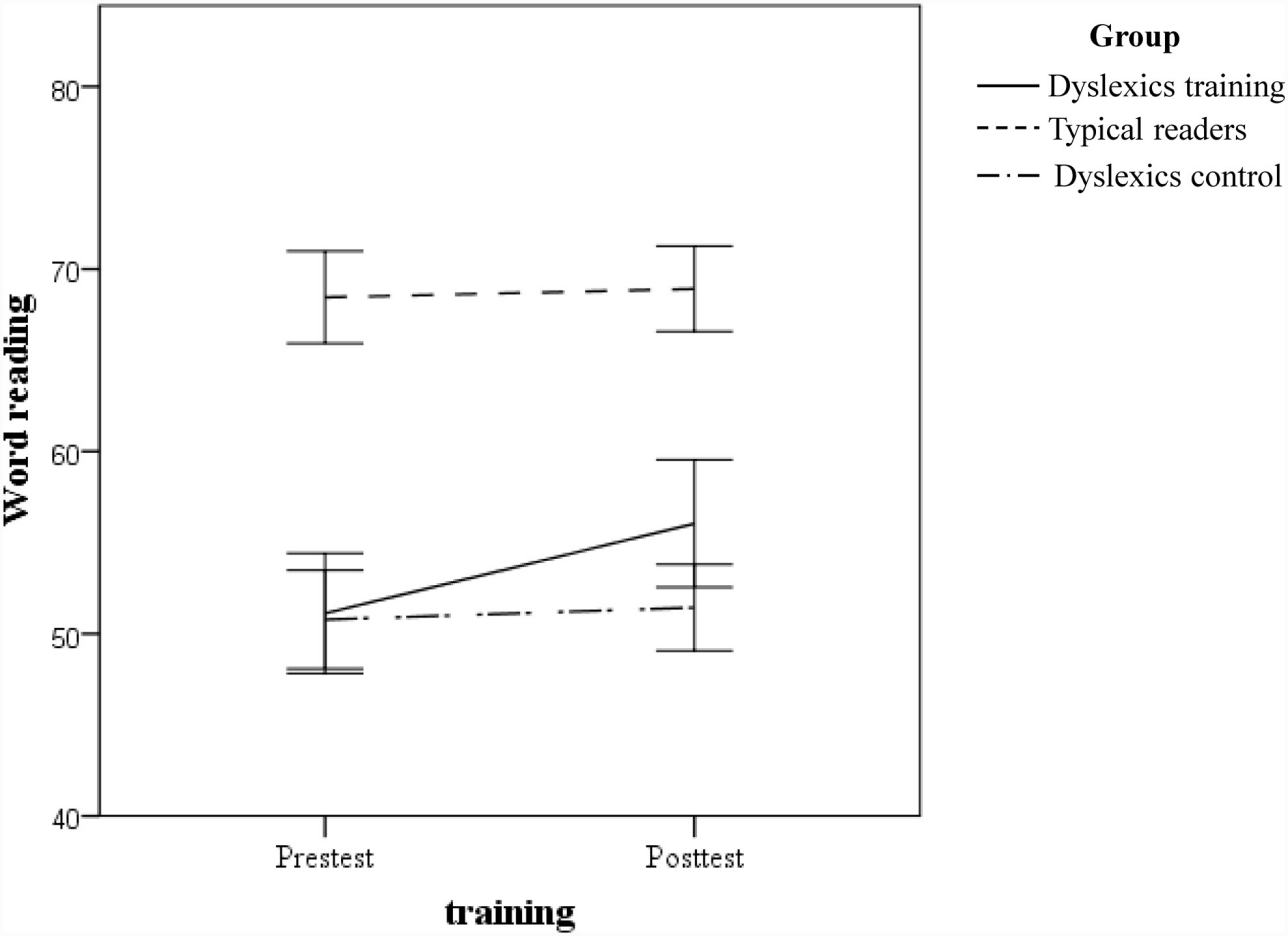
Group slopes for word reading. The figure displays the changes from pre- to post-test in the average of all test scores (accuracy and fluency) that loaded highly in the word-reading factor.

A mixed model with a random intercept and fixed factors Time and Group was performed. The t-test results in fixed effects estimates for the interaction between Group and Time are presented in Table 7. The results show that the slope of the word-reading factor associated with the training-group dyslexics was significantly different from that associated with typical readers, whereas the slopes did not differ between untrained dyslexics and typical readers. The slopes of the average of all the scores that loaded highly on the word-reading factor are plotted in Fig 3.

**Table 7:** Estimates of fixed effects for a random intercept model including time and group as fixed factors.

	Fixed effect estimates			
	Group—time interactions			
	DT : T		DC : T	
	*t*	*p-value*	*t*	*p-value*
Word reading	2.55	.013	0.45	.654
Mapping fluency	-1.18	.239	-0.08	.939
Mapping accuracy	0.70	.487	0.58	.566

T = typical readers; DT = dyslexics-training; DC = dyslexics control.

### Correlational analysis

An important aspect to consider when interpreting the training effects is that of the individual differences in reading gains. It is of interest to consider whether individual differences in letter-speech sound mapping are associated with variation in reading-fluency gains between pre- and post-test. Partial correlations were performed between the baseline factor scores associated with mapping fluency and post-test word reading fluency scores (controlled for pre-test differences in word reading fluency). The results are displayed in Table 8.

**Table 8:** Partial correlations with Letter-speech sound fluency factor score and post-test scores in reading fluency (pre-test controlled).

	Partial correlations with the factor LSS fluency					
	Typical readers		Dyslexics control		Dyslexics training	
	(N = 23)		(N = 21)^c^		(N = 23)^d^	
	*r*	*p*	*r*	*p*	*r*	*p*
3DM Word reading—*fluency* [T]^a^						
High Frequency	0.16	.468	-0.49	.037	0.39	.079
Low Frequency	0.07	.756	-0.42	.080	-0.01	.971
Pseudowords	-0.19	.403	-0.11	.650	-0.15	.504
Total	0.11	.632	-0.60	.008	0.03	.876
One-Minute Test—*fluency* [SS]^b^	0.41	.060	-0.59	.010	0.18	.431

^a^ T scores (M = 50, SD = 10).

^b^ SS scores (M = 10, SD = 3).

^c^ Valid cases for DC = 20 (attrition of one subject).

^d^ Valid cases for DT = 22.

Significant negative correlations between (baseline) mapping fluency and gains in reading fluency were found for waiting-list dyslexics for the main word reading scores, with the exception of low frequency word and pseudo-word reading scores. These results indicate that poorer initial mapping fluency is associated with lower gains in reading fluency in the untrained group. Significant correlations were absent for the typical readers group and further, initial mapping fluency was unrelated to reading fluency gains in the training dyslexics, i.e., participants in that group benefitted from the training regardless of their fluency in letter-speech sound mapping starting point. Notably, for both untrained dyslexics, trained dyslexics and typical readers, letter-speech sound mapping accuracy was not correlated with any of the reading fluency gains (all *r*’s between -0.31 and 0.18, *ps* > .210), except for a significant correlation between mapping accuracy and low frequency word scores (*r* = -0.53, *p* = .023; the poorer the initial accuracy, the higher the reading gain) in the untrained group. This result may indicate a less efficient identification of unfamiliar words for which effortful item-by-item decoding is required. But such an interpretation would be inconsistent with the apparent lack of a significant correlation between mapping accuracy and pseudowords, as the latter would arguably require similar decoding strategies as very low frequency words.

## Discussion

The present RCT study examines the beneficial effects of training letter-speech sound integration on reading fluency in 3^rd^ grade dyslexic readers. Groups were compared on a wide range of reading and letter-speech sound mapping measures. The latent factors derived from these measures were analyzed to evaluate training effects as well as differences in the rate of improvement between dyslexics and typical readers. Finally, the relationship between letter-speech sound mapping skills and reading improvement was examined in a correlational analysis. The results are interpreted within the framework of a letter-speech sound integration deficit in dyslexia.

### Baseline characteristics

Regarding the group comparisons at pre-test, the dyslexic groups showed a more severe impairment in word reading speed measures than in accuracy. This is consistent with previous research showing that in (semi-)transparent orthographies dyslexics may attain relatively high levels of reading accuracy after the first years of instructions while fluency is severely impaired [71–73]. In relation to letter-speech sound associations, the baseline group differences were less prominent than those of word reading. The pre-test group comparisons between dyslexics and typical readers revealed slightly larger effects on fluency than on accuracy scores, with the exception of the 3DM discrimination task, which was not sensitive to group differences in mapping fluency. A less pronounced deficit in mapping accuracy could be expected since children in 3^rd^ grade already present a reasonably advanced knowledge of letter-sound correspondences, even within poor readers. In addition, a previous study suggested that the letter-speech sound mapping accuracy deficit in dyslexics was absent after grade 2, while there was a halting of performance speed compared to typical readers in grade 3 [74]. According to the multisensory integration account, poor readers may know which letter correspond to which speech sound but still be unable to use these associations for fluent reading [17].

### Training effects on test scores

With regard to the remediation effects, the training-group dyslexics outperformed the waiting-group dyslexics after the letter-speech sound training program. The gains in word reading after training were more pronounced for fluency scores than for accuracy scores. Additionally, spelling scores and letter-speech sound identification fluency scores also showed improvement after training. The relatively small effects on word reading accuracy were anticipated given the high accuracy scores at pre-test. The effects of interventions for dyslexia on reading accuracy have been demonstrated in previous studies [49,75,76]. However, as argued in the introduction there is still a lack of robust evidence for effective treatments in terms of reading fluency. Interestingly, in the present study the largest effect sizes for gains after treatment were found in word reading fluency measures.

Most traditional intervention methods are based on phoneme awareness practice and phonemic decoding [3,49,77] which strongly focuses on the accurate learning of letter-speech sound correspondence rather than their automatic integration. In contrast, the present training aims to obtain automation of grapheme-phoneme mapping besides instruction and practice of accurate correspondences. The current results show that dyslexics are able to become more fluent readers by a systematic training in the automatisation of letters-speech sound correspondences. A potential confound might relate to group differences in the time between measurements. However, the inclusion of the time between measurements as a covariate did not change the pattern of results and the use of standardized scores controls for effects of time of reading instruction. Moreover, deficits in dyslexia have been shown to persist without special training [78], thus it seems unlikely that a few additional weeks of school attendance would have a significant impact on the observed differences between the dyslexic groups.

### Training effects manifested in factor scores

The results of the PCA analysis yielded three latent factors derived from the multiple outcome measures at pre-test; word reading, letter-speech sound mapping fluency and letter-speech sound mapping accuracy (see Table 4). The word reading measures of accuracy and speed accounted for the largest proportion of the variance, followed by letter-speech sound mapping fluency and accuracy, respectively. The reading speed measures also loaded on the other two factors. This may reflect that the contribution of letter-speech sound mapping skills to reading fluency is still relatively important in children in grade 3. This is supported by a previous study reporting moderate correlations between letter-speech sound identification and discrimination tasks and word reading tasks in transparent orthographies [79]. In addition, whereas letter-speech sound association scores of fluency and accuracy loaded highly on distinct factors, this was not the case for the word reading factor, which included both speed and accuracy scores.

The current finding of separate factors for fluency and accuracy of letter-speech sound associations has been reported in previous studies [74]. A potential confound may relate to the different response formats for fluency and accuracy (i.e., reaction time and proportion correct, respectively). But this confound would apply also to reading speed (indexed by the number of words) and accuracy (indexed by the percentage of words read correctly), both loading high on factor 1, which shared the highest loads. This pattern is in line with the notion that adequate knowledge of grapheme-phoneme correspondences does not necessarily lead to effective integration and fluent word reading [17]. With regard to the observed group differences, comparisons at pre-test revealed a clear difference between the two dyslexic groups vs. the typical readers for the three latent factor loadings. This was expected in view of the initial ANOVAs, suggesting that although the deficit in dyslexics was more prominent for word reading, their performance in letter-speech sound mapping tasks was also below the level of typical readers.

Most importantly, the analysis of training effects showed significant gains for the word reading factor in the training-group relative to the waiting-list group. The current training is exclusively focused on automatizing letter-speech sound mapping processes. These processes are essential for reading acquisition [23]. After training to develop more robust and automatic letter-speech sound associations, dyslexics may have been able to use these correspondences in a more efficient way for learning automatic word reading [25]. The gains in word reading after the current training further support the notion of a multisensory integration deficit underlying dyslexia [17,19,30]. Finally, the groups did not differ in gains in the two letter-speech sound mapping factors. Although there were differences present at test-level on letter-speech association tasks, the analysis failed to reveal statistical differences at the factor level. It could be possible that this lack is partially due to indifference of the behavioral letter-speech sound mapping measures [74] or insufficient statistical power in the present sample. In addition, the mapping fluency factor included the scores from letter-speech sound mapping discrimination task that, in our baseline comparison, failed to show a dyslexic deficit while the other tasks tapping mapping fluency, including spelling fluency and letter-speech sound identification, did show a moderate improvement after training in our analysis of test scores. Another plausible explanation would be that the current training improved reading fluency by other processes that are influenced by but not reflected in the letter-speech sound mapping tasks, such as visual word specialization. This is supported by the suggestion that the earlier development of grapheme-phoneme integration areas may support the later specialization of visual areas for fast recognition of words, which develops with increasing expertise in word reading [23,80,81].

### Rate of change

The rate of improvement for the word reading factor was faster in the training dyslexic group than in typical readers. Importantly, the rate of improvement for word reading did not differ between the control-group dyslexics vs. typical readers. Previous studies on normal reading development have indicated that while reading accuracy approaches ceiling levels after the first few years of instruction, reading fluency increase remains moderate over the years [74,82]. In view of this, low improvement in the word reading factor, which relates to both accuracy and speed measures, may be expected in typical readers in third grade, after attaining high fluency levels. The lack of differences between waiting-list dyslexics and typical readers suggests that severely deficient readers do not tend to catch up with those with higher reading skills. This is in line with previous longitudinal studies that have suggested stability in reading abilities. These studies found high correlations between reading scores across elementary grades [83–86]. The present results suggest that dyslexics do not overcome their deficit without special training. Moreover, the faster rate of change in training-group underscores the need for early and specialized intervention in dyslexia.

### Relation of reading fluency with mapping initial skills

The partial correlations suggested that reading fluency gains were related to baseline differences in letter-speech sound mapping fluency, in the waiting-list group but not in the training-group. This relation was absent in the typical readers group as well. Using the current longitudinal design, we show that in untrained dyslexics, reading fluency development is constrained by letter-speech sound association processes. This finding provides support for Blomert’s (2011) [17], suggestion that deficits in automatizing multisensory mapping may underlie reading dysfluency in dyslexia. Furthermore, this result supports the notion that training in automatizing letter-speech sound correspondences reduces integration deficits in reading fluency development. A possible interpretation of the current findings is that dyslexics at third grade might rely strongly on phonological decoding, similarly to typical readers during the initial stages of reading, unless specific training is provided [87].

### Limitations of the current study

There are two main limitations regarding the interpretation of current results. The first one relates to the design of the present study as only one type of intervention was tested. Consequently, the current design does not allow disentangling effects due to the specifics of the current training from those due to training in general training, such as increased reading exposure. Although this seems to be a common limitation in many intervention studies, reading dysfluency in dyslexia seems to persist even after specialized phonologically based interventions that can remediate accuracy problems [88]. Thus, the current improvements observed after a relatively short training are unlikely to be attributed to just increased reading exposure. A second limitation, related to the previous one, is concerned with the interpretation of our results based on the multisensory integration hypothesis. Obviously, our results offer only partial rather than decisive support for this hypothesis, as we did not find improvement in the dyslexics training group for the letter-speech sound mapping fluency factor. In addition, the deficits manifested in dyslexics in mapping fluency measures seemed to be less pronounced than in word reading. Previously, a study using these measures in a large sample of primary school children showed a decrease in response latencies until grade 5 in typical readers whereas in poor readers performance halted prematurely in grade 3 [60]. That study also found that accurate identification and discrimination of letter-speech sound pairs typically develops within the first year of instruction. Neuroimaging studies, however, showed a more prolonged period for the attainment of automatic integration at the neural level [39,89]. This observation may suggest that behavioral measures are not optimally sensitive to reveal the time demands of fully automatized multisensory integration. In this regard, apparent indifference of some of our behavioral measures may have influenced the specific patterning of the present results.

## Conclusions

The current RCT study demonstrates that a relative short but intensive training in letter-speech sound mapping fluency can significantly improve word reading in dyslexia. Importantly, the effects were not limited to reading accuracy skills; they also extended to reading fluency. The rate of improvement in the training-group was faster than both in typical readers and in dyslexics without special training. This is a promising result as reading fluency has repeatedly been shown to be unsusceptible to intervention in dyslexia [3,53,56]. Furthermore, reading fluency gains were strongly correlated to initial letter-speech sound mapping fluency in untrained dyslexics, suggesting that their reading fluency development is restricted by their mapping fluency. In contrast, reading fluency gains in the training group were unrelated to their initial mapping fluency. By systematically training fluency in grapheme-phoneme correspondences dyslexics thus seem to overcome their initial mapping deficiency barrier and are able to improve their reading fluency. This conclusion concurs with neurophysiological research showing that the ability to fluently integrate cross modal letter-speech sound information is critical for the development of a neural circuit for fast visual word recognition [17,33,90,91], as well as with reading development models in which the attainment of fluent letter-speech sound mappings are considered a critical step in the acquisition of fluent reading [23,92]. More specifically, reading research suggests that while children explicitly acquire initial knowledge of letter-speech sound mappings, the consequent implicit, statistical learning of grapheme-phoneme associations by repeated exposure drives the development towards the automatic integration of these mappings and their instrumental use in fluent reading [92–94]. Our results in accordance with this view, and thus suggest that intensive training towards automation of letter-speech sound integration is an important remedial activity in addressing reading fluency in dyslexia. At the same time, one might argue that these results provide an explanation for why interventions focusing essentially on phoneme awareness and decoding skills fail to improve reading fluency (e.g., [56]), as they bolster letter-speech sound mapping accuracy but do not intensively address the automation of letter-speech sound integration processes.

Recent neurophysiological and neuroanatomical studies have shown a deficit in the crossmodal integration of letters and speech sounds in a temporo-parietal network in dyslexia [17,20–22,91]. Notably, this deviant processing of letters and speech sounds in these multisensory areas has been reported in dyslexic children even if they attained adequate knowledge of letter-speech sound correspondences [30,39]. Based on these brain findings, a theoretical account of dyslexia has been postulated that states that a failure to develop automatic letter-speech sound integration will first and for all result in an impairment in the acquisition of fluent reading skills [17]. Using a behavioral intervention paradigm, we provided support for this account by showing that (a) accuracy in knowledge of letter-speech sound correspondences was not associated with reading fluency gains, (b) letter-speech sound mapping fluency was strongly correlated with fluency gains in untrained dyslexics, but not in trained dyslexics, and (c) an intensive training addressing the automation of letter-speech sound mappings produced reading fluency improvements.

Attaining reading fluency is a long process and previous studies have shown that even non-impaired readers may take years to become fluent readers [74]. The present results, together with those reported in Aravena et al. (2013) [43], illustrate the clinical potential of the letter-speech sound mapping framework for remediation programs in dyslexia.

## Supporting Information

S1 Checklist (DOC)

S1 Fig Touchscreen used in the training. (TIFF)

S1 Protocol (PDF)

## References

[1] Snowling MJ. Early identification and interventions for dyslexia: a contemporary view. J Res Spec Educ Needs. 2013;13:7–14. 10.1111/j.1471-3802.2012.01262.x 26290655 PMC4538781

[2] Blomert L. Dyslexie in Nederland. Amsterdam Uitg Nieuwezijds. 2005; Available: http://www.boomtestuitgevers.nl/upload/Dyslexie_in_Nederland_Leo_Blomert.pdf

[3] Gabrieli JDE. Dyslexia: a new synergy between education and cognitive neuroscience. Science. 2009;325:280–3. 10.1126/science.1171999 19608907

[4] Shaywitz SE, Shaywitz BA. Paying attention to reading: the neurobiology of reading and dyslexia. Dev Psychopathol. 2008;20:1329–49. 10.1017/S0954579408000631 18838044

[5] UNESCO. Education for all: Literacy for life. Paris: UNESCO publishing; 2005.

[6] Vellutino FR, Fletcher JM, Snowling MJ, Scanlon DM. Specific reading disability (dyslexia): what have we learned in the past four decades? J Child Psychol Psychiatry. 2004;45:2–40. Available: http://www.ncbi.nlm.nih.gov/pubmed/14959801 14959801 10.1046/j.0021-9630.2003.00305.x

[7] Bishop DVM. Dyslexia: What’s the problem? Dev Sci. 2006;9:256–257. 16669792 10.1111/j.1467-7687.2006.00484.x

[8] Blomert L, Willems G. Is there a causal link from a phonological awareness deficit to reading failure in children at familial risk for dyslexia? Dyslexia. 2010;16:300–317. 20957685 10.1002/dys.405

[9] Castles A, Coltheart M. Is there a causal link from phonological awareness to success in learning to read? Cognition. 2004;91:77–111. 10.1016/S0010-0277(03)00164-1 14711492

[10] Castles A, Coltheart M, Wilson K, Valpied J, Wedgwood J. The genesis of reading ability: what helps children learn letter-sound correspondences? J Exp Child Psychol. Elsevier Inc.; 2009;104:68–88. 10.1016/j.jecp.2008.12.003 19268301

[11] Boets B, Smedt B, Cleuren L, Vandewalle E, Wouters J, Ghesquière P. Towards a further characterization of phonological and literacy problems in Dutch-speaking children with dyslexia. Br J Dev Psychol. 2010;28:5–31. 10.1348/026151010X485223 20306623

[12] Mann V, Wimmer H. Phoneme awareness and pathways into literacy : A comparison of German and American children. Read Writ. 2002;15:653–682.

[13] Morais J, Castro SL, Scliar-Cabral L, Kolinsky R, Content A. The effects of literacy on the recognition of dichotic words. Q J Exp Psychol. 1987;39:451–465. 10.1080/14640748708401798 3671762

[14] Dehaene S, Pegado F, Braga LW, Ventura P, Nunes Filho G, Jobert A, et al How learning to read changes the cortical networks for vision and language. Science (80-). 2010;330:1359–64. 10.1126/science.1194140 21071632

[15] Molfese DL. Predicting dyslexia at 8 years of age using neonatal brain responses. Brain Lang. 2000;72:238–245. 10.1006/brln.2000.2287 10764519

[16] Biancarosa G, Snow C. Reading next: A vision for action and research in middle and high school literacy: A report from Carnegie Corporation of New York. 2nd ed Washington, DC: Alliance for Excellent Education; 2004.

[17] Blomert L. The neural signature of orthographic-phonological binding in successful and failing reading development. Neuroimage. 2011;57:695–703. 10.1016/j.neuroimage.2010.11.003 21056673

[18] Maurer U, Blau V, Yoncheva YN, McCandliss BD. Development of Visual Expertise for Reading : Rapid Emergence of Visual Familiarity for an Artificial Script. Dev Neuropsychol. 2010;35:404–422. 20614357 10.1080/87565641.2010.480916 PMC3008655

[19] Froyen D, Willems G, Blomert L. Evidence for a specific cross-modal association deficit in dyslexia: an electrophysiological study of letter-speech sound processing. Dev Sci. 2011;14:635–48. 10.1111/j.1467-7687.2010.01007.x 21676085

[20] Kronschnabel J, Brem S, Maurer U, Brandeis D. The level of audiovisual print-speech integration deficits in dyslexia. Neuropsychologia. Elsevier; 2014;62:245–261. 10.1016/j.neuropsychologia.2014.07.024 25084224

[21] Wallace MT. Dyslexia: bridging the gap between hearing and reading. Curr Biol. Elsevier Ltd; 2009;19:R260–2. 10.1016/j.cub.2009.01.025 PMC4013749 19321145

[22] Žarić G, Fraga González G, Tijms J, van der Molen MW, Blomert L, Bonte M. Reduced neural integration of letters and speech sounds in dyslexic children scales with individual differences in reading fluency. PLoS One. 2014;9:e110337 10.1371/journal.pone.0110337 25329388 PMC4199667

[23] Ehri LC. Learning to Read Words: Theory, Findings, and Issues. Scientific Studies of Reading. 2005 pp. 167–188. 10.1207/s1532799xssr0902_4

[24] Frith U. Beneath the surface of developmental dyslexia Patterson K E, Marshall J C, & Coltheart M (Eds)Surface dyslexia. London:Erlbaum; 1985 pp. 301–330.

[25] Ehri LC, Saltmarsh J. Beginning readers outperform older disabled readers in learning to read words by sight. Read Writ. 1995;7:295–326. 10.1007/BF03162082

[26] Breznitz Z, Berman L. The Underlying Factors of Word Reading Rate. Educ Psychol Rev. 2003;15:247–265. 10.1023/A:1024696101081

[27] Meyler A, Breznitz Z. Visual, auditory and cross-modal processing of linguistic and nonlinguistic temporal patterns among adult dyslexic readers. Dyslexia. 2005;11:93–115. 10.1002/dys.294 15918369

[28] Raij T, Uutela K, Hari R. Audiovisual integration of letters in the human brain. Neuron. 2000;28:617–25. Available: http://www.ncbi.nlm.nih.gov/pubmed/11144369 11144369 10.1016/s0896-6273(00)00138-0

[29] Hashimoto R, Sakai KL. Learning letters in adulthood: direct visualization of cortical plasticity for forming a new link between orthography and phonology. Neuron. 2004;42:311–22. Available: http://www.ncbi.nlm.nih.gov/pubmed/15091345 15091345 10.1016/s0896-6273(04)00196-5

[30] Blau V, Reithler J, van Atteveldt N, Seitz J, Gerretsen P, Goebel R, et al Deviant processing of letters and speech sounds as proximate cause of reading failure: a functional magnetic resonance imaging study of dyslexic children. Brain. 2010;133:868–879. 10.1093/brain/awp308 20061325

[31] Van Atteveldt N, Formisano E, Goebel R, Blomert L. Integration of letters and speech sounds in the human brain. Neuron. 2004;43:271–82. 10.1016/j.neuron.2004.06.025 15260962

[32] McCandliss BD, Noble KG. The development of reading impairment: a cognitive neuroscience model. Ment Retard Dev Disabil Res Rev. 2003;9:196–204. 10.1002/mrdd.10080 12953299

[33] Sandak R, Mencl W, Frost SJ, Pugh KR. The Neurobiological Basis of Skilled and Impaired Reading : Recent Findings and New Directions. Sci Stud Read. 2004;8:273–292.

[34] Brunswick N, McCrory E, Price C, Frith C, Frith U. Explicit and implicit processing of words and pseudowords by adult developmental dyslexics A search for Wernicke’s Wortschatz? Brain. 1999;122:1901–1917. Available: http://brain.oxfordjournals.org/content/122/10/1901.short 10506092 10.1093/brain/122.10.1901

[35] Helenius P, Tarkiainen A, Cornelissen PL, Hansen PC, Salmelin R. Dissociation of normal feature analysis and deficient processing of letter-strings in dyslexic adults. Cereb cortex. 1999;9:476–83. Available: http://www.ncbi.nlm.nih.gov/pubmed/10450892 10450892 10.1093/cercor/9.5.476

[36] Paulesu E, Démonet JF, Fazio F, McCrory E, Chanoine V, Brunswick N, et al Dyslexia: cultural diversity and biological unity. Science (80-). 2001;291:2165–7. 10.1126/science.1057179 11251124

[37] Simos P, Breier JI, Fletcher JM, Bergman E, Papanicolaou a C. Cerebral mechanisms involved in word reading in dyslexic children: a magnetic source imaging approach. Cereb cortex. 2000;10:809–16. Available: http://www.ncbi.nlm.nih.gov/pubmed/10920052 10920052 10.1093/cercor/10.8.809

[38] Fraga González G, Žarić G, Tijms J, Bonte M, Blomert L, van der Molen MW. Brain-potential analysis of visual word recognition in dyslexics and typically reading children. Front Hum Neurosci. 2014;8:1–14. 25071507 10.3389/fnhum.2014.00474 PMC4075352

[39] Froyen D, Bonte M, van Atteveldt N, Blomert L. The long road to automation: neurocognitive development of letter—speech sound processing. J Cogn Neurosci. 2009;21:567–580. Available: http://www.mitpressjournals.org/doi/abs/10.1162/jocn.2009.21061 10.1162/jocn.2009.21061 18593266

[40] Blau V, van Atteveldt N, Ekkebus M, Goebel R, Blomert L. Reduced neural integration of letters and speech sounds links phonological and reading deficits in adult dyslexia. Curr Biol. Elsevier Ltd; 2009;19:503–8. 10.1016/j.cub.2009.01.065 19285401

[41] Raschle NM, Stering PL, Meissner SN, Gaab N. Altered Neuronal Response During Rapid Auditory Processing and Its Relation to Phonological Processing in Prereading Children at Familial Risk for Dyslexia. Cereb cortex. 2013;2489–2501. 10.1093/cercor/bht104 PMC4184369 23599167

[42] Lyytinen H, Guttorm TK, Huttunen T, Hämäläinen J, Leppänen PHT, Vesterinen M. Psychophysiology of developmental dyslexia: a review of findings including studies of children at risk for dyslexia. J Neurolinguistics. 2005;18:167–195. 10.1016/j.jneuroling.2004.11.001

[43] Aravena S, Snellings P, Tijms J, van der Molen MW. A lab-controlled simulation of a letter-speech sound binding deficit in dyslexia. J Exp Child Psychol. 2013;115:691–707. 10.1016/j.jecp.2013.03.009 23708733

[44] Tijms J. The Development of Reading Accuracy and Reading Rate during Treatment of Dyslexia. Educ Psychol. 2007;27:273–294. 10.1080/01443410601066800

[45] Hatcher PJ, Hulme C, Miles JN V, Carroll JM, Hatcher J, Gibbs S, et al Efficacy of small group reading intervention for beginning readers with reading-delay: a randomised controlled trial. J Child Psychol Psychiatry. 2006;47:820–7. 10.1111/j.1469-7610.2005.01559.x 16898996

[46] Lovett M., Barron R., Benson NJ. Effective remediation of word identification and decoding difficulties in school-age children with reading disabilities. Handb Learn Disabil. 2003;11:273–292.

[47] National Reading Panel. Teaching children to read: An evidence based assessment of the scientific research literature on reading and its implication for reading instruction: Reports of the subgroups. Washington, DC: National Institute of Child Health and Human Development, National Institutes of Health; 2000.

[48] Thaler V, Ebner EM, Wimmer H, Landerl K. Training reading fluency in dysfluent readers with high reading accuracy: word specific effects but low transfer to untrained words. Ann Dyslexia. 2004;54:89–113. Available: http://www.ncbi.nlm.nih.gov/pubmed/15765005 15765005 10.1007/s11881-004-0005-0

[49] Alexander AW, Slinger-Constant A-M. Current status of treatments of dyslexia: Critical review. J Child Neurol. 2004;19:744–758. 15559890 10.1177/08830738040190100401

[50] Levy B. Moving to the bottom: Improving reading fluency. Dyslexia, fluency, and the brain. 2001;357–379.

[51] Chard DJ, Vaughn S, Tyler B-J. A Synthesis of Research on Effective Interventions for Building Reading Fluency with Elementary Students with Learning Disabilities. J Learn Disabil. 2002;35:386–406. 10.1177/00222194020350050101 15490537

[52] Eden GF, Moats L. The role of neuroscience in the remediation of students with dyslexia. Nat Neurosci. 2002;5:1080–1084. 10.1038/nn946 12403991

[53] Compton DL, Miller AC, Elleman AM, Steacy LM. Have We Forsaken Reading Theory in the Name of “Quick Fix” Interventions for Children With Reading Disability? Sci Stud Read. 2014;18:55–73.

[54] Torgesen JK, Alexander AW, Wagner RK, Rashotte CA, Voeller KKS, Conway T. Intensive remedial instruction for children with severe reading disabilities immediate and long-term outcomes from two instructional approaches. J Learn Disabil. 2001;34:33–58. 10.1177/002221940103400104 15497271

[55] Kuhn MR, Stahl S a. Fluency: A review of developmental and remedial practices. J Educ Psychol. 2003;95:3–21.

[56] Elliott JG, Grigorenko EL. The dyslexia debate. Cambridge University Press; 2014.

[57] Raven JC, Court JH. Coloured progressive matrices. Oxford, UK: Oxford Psychologists Press; 1998.

[58] Achenbach TM, McConaughy SH. The Achenbach System of Empirically Based Assessment (ASEBA) Handbook of psychological and educational assessment of children. New York, NY: Guilford Press; 2003 pp. 406–430.

[59] Borenstein M, Rothstein H, Cohen J. Power and precision TM. Englewood, NJ: Biostat, Inc.; 2001.

[60] Blomert L, Vaessen A. 3DM differential diagnostics for dyslexia: cognitive analysis of reading and spelling. Amsterdam, the Netherlands: Boom Test; 2009.

[61] Baayen RH, Piepenbrock R, Gulikers L. The CELEX lexical database. 1995;

[62] Van den Bos KP, Spelberg L, Scheepsma AJM, De Vries JR. De Klepel: Verantwoording, handleiding, Diagnostiek en behandeling. Lisse, the Netherlands: Swets & Zeitlinger; 1999.

[63] De Vos T. Schoolvaardigheidstoets. Amsterdam, the Netherlands: Boom Test; 2007.

[64] Davydov VV. The state of research on learning activity. J Russ East Eur Psychol. 1995;33:55–70.

[65] Schneider W. Controlled & automatic processing: behavior, theory, and biological mechanisms. Cogn Sci. 2003;27:525–559.

[66] Grigorenko EL. Developmental dyslexia: an update on genes, brains, and environments. J Child Psychol Psychiatry. 2001;42:91–125. Available: http://www.ncbi.nlm.nih.gov/pubmed/11205626 11205626

[67] Legge GE, Mansfield JS, Chung ST. Psychophysics of reading. XX. Linking letter recognition to reading speed in central and peripheral vision. Vision Res. 2001;41:725–43. Available: http://www.ncbi.nlm.nih.gov/pubmed/11248262 11248262 10.1016/s0042-6989(00)00295-9

[68] Van Breukelen GJP. ANCOVA versus change from baseline: more power in randomized studies, more bias in nonrandomized studies. J Clin Epidemiol. 2006;59:920–5. 16895814 10.1016/j.jclinepi.2006.02.007

[69] Thompson B. Exploratory and confirmatory factor analysis: Understanding concepts and applications. Am Psychol Assoc 2004;

[70] Snijders TAB, Bosker RJ. Multilevel analysis: An introduction to basic and advanced multilevel modelling. London: Sage; 1999.

[71] De Jong PF, van der Leij A. Developmental changes in the manifestation of a phonological deficit in dyslexic children learning to read a regular orthography. J Educ Psychol. 2003;95:22–40.

[72] Yap R, Leij A. Word processing in dyslexics. Read Writ. 1993;5:261–279. 10.1007/BF01027391

[73] Landerl K, Wimmer H, Frith U. The impact of orthographic consistency on dyslexia: a German-English comparison. Cognition. 1997;63:315–34. Available: http://www.ncbi.nlm.nih.gov/pubmed/9265873 9265873 10.1016/s0010-0277(97)00005-x

[74] Vaessen A, Blomert L. Long-term cognitive dynamics of fluent reading development. J Exp Child Psychol. Elsevier Inc.; 2010;105:213–31. 10.1016/j.jecp.2009.11.005 20042196

[75] Galuschka K, Ise E, Krick K, Schulte-Körne G. Effectiveness of treatment approaches for children and adolescents with reading disabilities: a meta-analysis of randomized controlled trials. PLoS One. 2014;9:e89900 10.1371/journal.pone.0089900 24587110 PMC3935956

[76] Tijms J. Effectiveness of computer-based treatment for dyslexia in a clinical care setting: outcomes and moderators. Educ Psychol. 2011;31:873–896. Available: http://www.tandfonline.com/doi/abs/10.1080/01443410.2011.621403

[77] Wolff U. Effects of a randomised reading intervention study: an application of structural equation modelling. Dyslexia. 2011;17:295–311. 10.1002/dys.438 21953739

[78] Snowling MJ, Muter V, Carroll J. Children at family risk of dyslexia: a follow-up in early adolescence. J Child Psychol Psychiatry. 2007;48:609–18. 10.1111/j.1469-7610.2006.01725.x 17537077

[79] Vaessen A, Bertrand D, Tóth D, Csépe V, Faísca L, Reis A, et al Cognitive development of fluent word reading does not qualitatively differ between transparent and opaque orthographies. J Educ Psychol. 2010;102:827–842.

[80] McCandliss BD, Cohen L, Dehaene S. The visual word form area: expertise for reading in the fusiform gyrus. Trends Cogn Sci. 2003;7:293–299. 10.1016/S1364-6613(03)00134-7 12860187

[81] Pugh KR, Landi N, Preston JL, Mencl W, Austin AC, Sibley D, et al The relationship between phonological and auditory processing and brain organization in beginning readers. Brain Lang. Elsevier Inc.; 2013;125:173–83. 10.1016/j.bandl.2012.04.004 PMC3417084 22572517

[82] Wimmer H, Hummer P. How German-speaking first graders read and spell: Doubts on the importance of the logographic stage. Appl Psycholinguist. 1990;11:349–368.

[83] Juel C. Learning to read and write: A longitudinal study of 54 children from first through fourth grades. J Educ Psychol. 1988;80:437–447.

[84] Wagner RK, Torgesen JK, Rashotte C a, Hecht S a, Barker T a, Burgess SR, et al Changing relations between phonological processing abilities and word-level reading as children develop from beginning to skilled readers: a 5-year longitudinal study. Dev Psychol. 1997;33:468–79. Available: http://www.ncbi.nlm.nih.gov/pubmed/9149925 9149925 10.1037//0012-1649.33.3.468

[85] Aunola K, Nurmi N, Niemi P, Lerkannen MK, Rasku-Puttonen H. Developmental dynamics of achievement strategies, reading performance, and parental beliefs. Read Res Q. 2002;37:310–327.

[86] Parrila R, Kirby J, McQuarrie L. Articulation rate, naming speed, verbal short-term memory, and phonological awareness: Longitudinal predictors of early reading development? Sci Stud Read. 2004;8:3–26.

[87] Maurer U, Schulz E, Brem S, der Mark S Van, Bucher K, Martin E, et al The development of print tuning in children with dyslexia: evidence from longitudinal ERP data supported by fMRI. Neuroimage. Elsevier Inc.; 2011;57:714–22. 10.1016/j.neuroimage.2010.10.055 21040695

[88] Shaywitz SE, Morris R, Shaywitz BA. The education of dyslexic children from childhood to young adulthood. Annu Rev Psychol. 2008;59:451–475. 18154503 10.1146/annurev.psych.59.103006.093633

[89] Booth JR, Burman DD, Van Santen FW, Harasaki Y, Gitelman DR, Parrish TB, et al The development of specialized brain systems in reading and oral-language. Child Neuropsychol. 2001;7:119–141. 10.1076/chin.7.3.119.8740 12187470

[90] Gullick M, Booth J. Individual differences in crossmodal brain activity predict arcuate fasciculus connectivity in developing readers. J Cogn Neurosci. 2014;26:1331–1346. 24456399 10.1162/jocn_a_00581 PMC4828929

[91] Hahn N, Foxe JJ, Molholm S. Impairments of multisensory integration and cross-sensory learning as pathways to dyslexia. Neurosci Biobehav Rev. Elsevier Ltd; 2014;47:384–392. 10.1016/j.neubiorev.2014.09.007 PMC4258132 25265514

[92] Gombert J. Implicit and explicit learning to read: Implication as for subtypes of dyslexia. Curr Psychol Lett. 2003;10:1–7. Available: http://cpl.revues.org/202?type=motcle

[93] Aravena S, Tijms J. Reading Fluency and dyslexia: innovative developments in the role of associative learning and repetitive exposure in skill acquisition In: In Larson J. E. (Ed.), editor. Educational Psychology: Cognition and learning, individual differences, and motivation. Hauppage, NY: Nova Science; 2009 pp. 113–141.

[94] Pavlidou E V, Williams JM. Implicit learning and reading: insights from typical children and children with developmental dyslexia using the artificial grammar learning (AGL) paradigm. Res Dev Disabil. Elsevier Ltd.; 2014;35:1457–72. 10.1016/j.ridd.2014.03.040 24751907

