# Supplementary figures and images for: A Randomized Controlled Trial on The Beneficial Effects of Training Letter-Speech Sound Integration on Reading Fluency in Children with Dyslexia
Source: PLoS One. 2015 Dec 2;10(12):e0143914. doi: 10.1371/journal.pone.0143914 (PMC4667884; doi:10.1371/journal.pone.0143914)

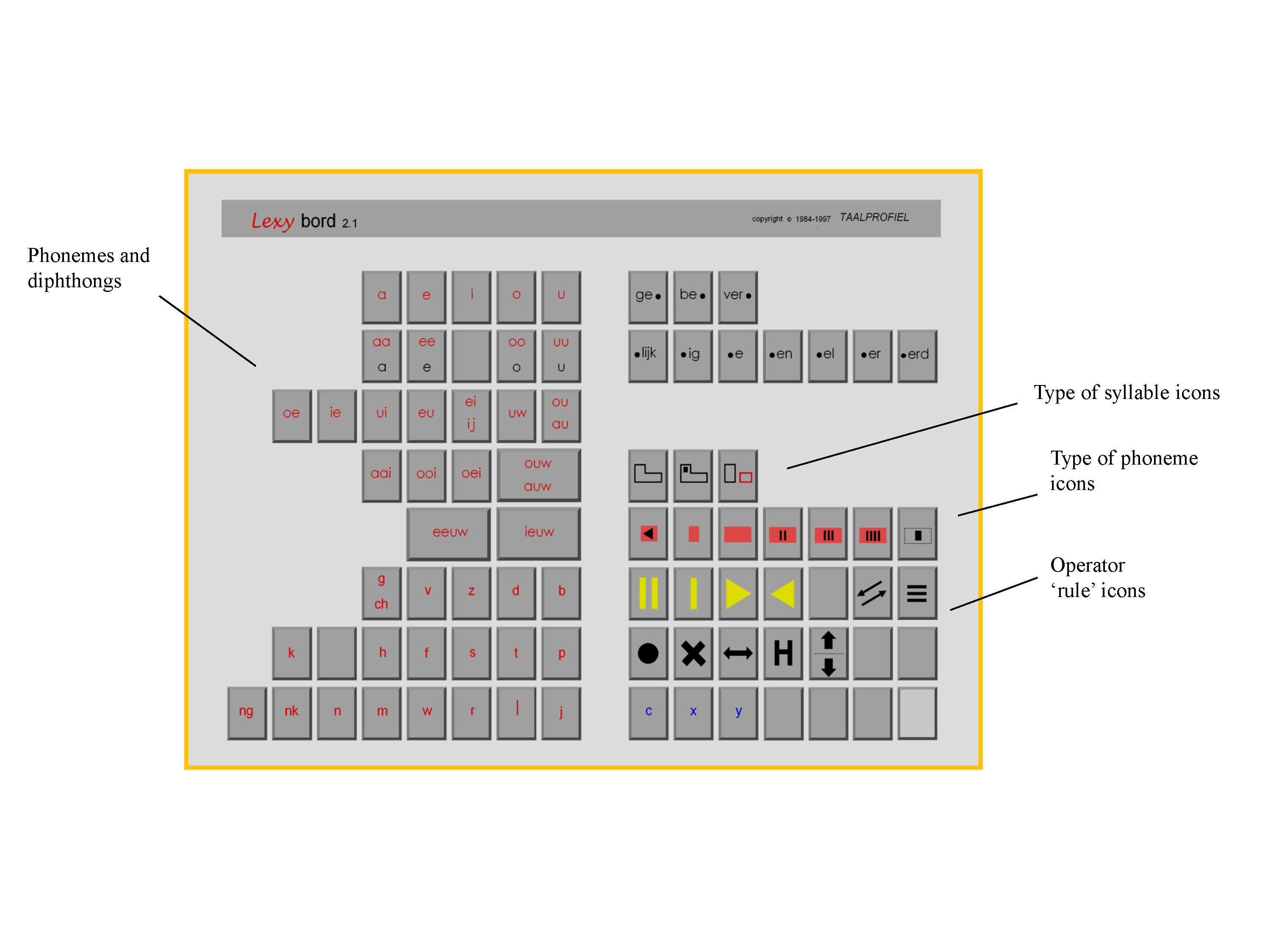

Supplement: S1 Fig — (TIFF) [file pone.0143914.s002.tiff]
