# Supplementary material for: A Randomized Controlled Trial on The Beneficial Effects of Training Letter-Speech Sound Integration on Reading Fluency in Children with Dyslexia
Source: PLoS One. 2015 Dec 2;10(12):e0143914. doi: 10.1371/journal.pone.0143914 (PMC4667884; doi:10.1371/journal.pone.0143914)
Supplement: S1 Protocol — (PDF) [file pone.0143914.s003.pdf]

UNIVERSITEIT VAN AMSTERDAM

Universiteit Maastricht

PROTOCOL

Training letter-speech sound associations to improve reading fluency in children with dyslexia

1. RESEARCH TEAM

Prof. dr. L. Blomert, Maastricht University
Prof. dr. M. W. van der Molen, University of Amsterdam
Dr. J. Tijms, University of Amsterdam
Dr. M. Bonte, Maastricht University
G. Fraga González, University of Amsterdam
G. Žarić, Maastricht University

2. BACKGROUND

Successful interventions in dyslexia show that reasonable levels of reading accuracy may be attainable (e.g. Tijms et al, 2003; Tijms, 2007; Torgesen, 2005), but no effective cure for the lack of reading fluency is available yet. This may in part be attributed to the fact that the dominant theory of dyslexia assumes a single main cause, a phonological awareness deficit, which never offered a valid explanation for the persistent dysfluency in dyslexia (Blomert & Willems, 2010), but nevertheless inspired and still inspires most reading interventions. A very recent account therefore proposed a core multi-sensory integration deficit in a temporo-parietal network, almost simultaneously affecting superior temporal areas involved in speech processing and fusiform areas involved in visual word recognition (Blomert, in revision). Relevant for present purposes, this new theoretical account also predicts that " a failure to develop automatic letter-sound integration will first and for all result in an impairment of reading fluency" (Blomert, in revision; Blomert & Froyen, 2010).

Goal of the study is improving reading fluency in dyslexia. We will therefore deliver an intervention focussed on developing of automatic letter-speech sound integration to children with dyslexia, in order to provide a neurocognitive window on the learning mechanisms involved in developing reading fluency.

3. STUY OBJECTIVES

The main objective of study is to determine whether a computer-based intervention focussed on intensively training automation of letter-speech-sound associations can improve reading fluency in children with severe dyslexia.

In more detail, our objectives are:
- Does the intervention improve knowledge of letter-speech sound associations, compared to the no-intervention control group?
- Does the intervention improve automation of letter-speech sound integration, compared to no-intervention control group?
- Does the intervention improve reading accuracy, compared to the no-intervention control group?
- Does the intervention improve reading fluency, compared to the no-intervention control group?
- Are the gains in reading fluency associated with initial levels and/or gains in the automation of letter-speech sound associations?
- Are the intervention-induced reading-related growth curves of the dyslexic children comparable to those of normal readers?
- Does the intervention induce changes in brain responses in a local network related to letter-speech sound processing, as measured by ERPs of audiovisual MMN?
- Does the intervention induce changes in brain responses in a more global neural network for fast visual word processing, as measured by ERPs of visual word recognition?

4. RESEARCH METHODS

4.1. Study Design

The study is an open randomised controlled trial comparing an intervention addressing letter-speech sound integration to a waiting list control group. Children with severe dyslexia are randomly assigned to either an immediate intervention group or a waiting-list control group by a method of simple randomisation. Participants will be randomised using a computerised random number generator. Children in the intervention condition will receive 34 intervention-sessions in a 19 week period.

In addition, a second control group of normal readers who do not receive the intervention (same age-range, school grade, sociodemographical background, and exclusion criteria as the children with dyslexia) is included in the study.

Behavioural measures of reading, spelling, and letter-speech sound mapping are measured at baseline and at the end of a 19 week (intervention) period for all three groups. EEG measurements of visual word recognition and of letter-speech sound integration are measured at baseline and at the end of the intervention (after 19 weeks) for the intervention group, and only once, at approximately 10 weeks, for both the waiting-list control group and the normal-reader control group (used as a criterion point to compare the pre-post ERP-changes in the intervention with).

4.2. Subject Selection

Inclusion Criteria for severe dyslexia

- Diagnosis of dyslexia (according to criteria of Dutch Dyslexia Foundation, and DSM-IV-TR (reading disorder))
- Suffering from specific and persistent (i.e., failed to respond to remedial support at school) problems with reading at school
- Percentile score ≤ 10% on a standard reading test
- Age 8-9 years old
- In grade 3 of primary education

Inclusion Criteria for normal readers

- No diagnosis of dyslexia
- Percentile score ≥ 25% on standard reading test
- Age 8-9 years old
- In grade 3 of primary education
- In the same sample of schools as where the dyslexic children are referred from

Exclusion Criteria (for all participants)

- Behavioral and/or attention disorders, as measured by the Child Behavior Checklist (CBCL)
- IQ < 85
- Uncorrected sight problems
- Hearing loss

4.3. Number of Participants

Planned sample size: N = 50 children with severe dyslexia; subjects fulfilling the entry criteria will be randomised to one of the two trial arms. N=25 normal reading children.

4.4. Subject Recruitment

Children with dyslexia will be recruited through the IWAL Institute. Children who (1) are referred by schools to the IWAL Institute because of specific and persistent reading problems at school, (2) received a diagnosis of dyslexia after an extensive cognitive assessment by a psychologist at the IWAL Institute, (3) had a reading score ≤ 10% on a standard reading test at the diagnostic assessment, and (4) were in grade 3 at school, are recruited.

Normal reading children will be recruited from the same sample of schools as the dyslexic children are referred from.

4.5. Informed Consent

All participants will receive a consent form. The consent form explains the aims of the study, and the study procedures. The consent form explicitly states that the participant is free to withdraw at any time without any need to give reason.

4.6. Sample size justification

Before running a sample size analysis, we first calculated the expected effect size for the intervention gains in reading fluency on the basis of the data of previous intervention studies (Tijms, in revision, Saine et al., 2009). These data revealed expected effect sizes in terms of Cohen's f in the range of 0.33 - 0.40 for gains in reading fluency by intervention in comparison to a no-intervention control group. This is generally considered a medium to large effect size. Therefore, our expected intervention effect size is $f = 0.33 – 0.40$.

As we intent to analyse the intervention effects by means of an ANCOVA with pretest scores as covariates for each corresponding posttest score (Vickers & Altman, 2001), we also calculated the R^2 for the covariate pretest score in reading fluency in the study of Tijms (in revision): $R^2 = .37$. By including an expected R^2 for the covariate, we were able to calculate the sample size more precisely.

Next, we run a sample size calculation (Power & Precision V4 software; Borenstein, Rothstein, & Cohen, 2001). Our aim is a power of at least 0.80 to detect an intervention effect on reading fluency, assuming the above noted expected effect size ($f = 0.33 - 0.40$), and conducting an ANCOVA with 1 factor (intervention condition, 2 levels) and 1 covariate (pretest level, $R^2 = .37$), and $\alpha = 0.05$. The power analysis revealed a power of at least 0.80 for $n \geq 20$ per intervention condition.

Based on this power calculation and given an anticipated attrition rate of 20%, we aim to recruit a total of 50 dyslexic children.

4.7. Intervention

The intervention in this study is a computerised intervention focussed on the integration of letters and speech sounds in the context of the acquisition of reading fluency. This training is an adaptation of the treatment protocol reported in Tijms (2007). The intervention program is delivered by well-instructed junior psychologists, on a one-to-one basis for 45-minute sessions during a 19 week period. The training frequency is two sessions per week. Intervention takes place at a clinical centre.

4.8. Intervention Sites

This intervention will be conducted at the Rudolf Berlin Center in Amsterdam, and at the associated locations of the IWAL Institute in the Amsterdam area.

4.9. Measures

Behavioral measures

Baseline and posttest:
Reading
Word Reading – Accuracy, 3DM (Blomert & Vaessen, 2009)
Word Reading – Fluency, 3DM (Blomert & Vaessen, 2009)
Word Reading Fluency, EMT (Brus & Voeten, 1999)
Text Reading Fluency, SVT-TL (De Vos, 2007)

Spelling
Spelling – Accuracy, 3DM (Blomert & Vaessen, 2009)
Spelling – Fluency, 3DM (Blomert & Vaessen, 2009)

Letter-Speech Sound Mapping
Letter-Speech Sound Identification – Accuracy, 3DM (Blomert & Vaessen, 2009)
Letter-Speech Sound Identification – Fluency, 3DM (Blomert & Vaessen, 2009)
Letter-Speech Sound Discrimination – Accuracy, 3DM (Blomert & Vaessen, 2009)
Letter-Speech Sound Discrimination – Fluency, 3DM (Blomert & Vaessen, 2009)

Baseline only:
Child Behavior Checklist (CBCL, Achenbach & McConaughy, 2003)
Coloured Progressive Matrices (Raven, Raven, & Court, 1998)

Behavioural measures are tested individually at either the clinical centre for the dyslexic children or at school for the normal readers during one session of approximately one hour. Children are tested in a silent room. The Child Behavior Checklist is completed by parents via a web-based form.

EEG measures

- ERP (Event-Related Potential), audiovisual MMN of letter-speech sound pairs
- ERP, visual word recognition

The EEG protocol is provided in Appendix A.

4.10. Timeline

Recruitment of participants: October – December 2011
Testing and intervention: December 2011 – July 2012

See Appendix B for a more detailed timeline.

5. FUNDING

This project is funded by the Netherlands Initiative Brain and Cognition, a program of the Organization for Scientific Research (NWO), as part of the project "Fluent reading acquisition neurocognitively decomposed: The case of dyslexia" under grant number 056-14-015.

6. REFERENCES

Achenbach, T. M., & McConaughy, S. H. (2003). The Achenbach System of Empirically Based Assessment (ASEBA). In C. R. Reynolds, & R. W. Kamphaus (Eds.), *Handbook of psychological and educational assessment of children* (pp. 406–430). New York, NY: Guilford Press.

Blomert, L. (in revision). The neural signature of orthographic-phonological binding in successful and failing reading development.

Blomert, L., & Froyen, D. (2010). Multi-sensory learning and learning to read. International *Journal of Psychophysiology, 77,* 195-204.

Blomert, L., & Vaessen, A. (2009). *3DM: Differentiaal diagnostiek van dyslexie.* Amsterdam: Boom Test Publishers.

Blomert, L., & Willems, G. (2010). Is there a causal link from a phonological awareness deficit to reading failure in children with familial risk for dyslexia. *Dyslexia, 16,* 300-317.

Borenstein, M., Rothstein, H., & Cohen, J. (2001). *Power and Precision*. Englewood, NJ: Biostat.

De Vos, T. (2007). *Schoolvaardigheidstoets technisch lezen*. Amsterdam: Boom test Publishers.

Saine, N. L., Lerkkanen, M.-J., Ahonen, T., Tolvanen, A., & Lyytinen, H. (2010). Predicting word-level reading fluency outcomes in three contrastive groups: Remedial and computer-assisted remedial reading intervention, and mainstream instruction. *Learning and Individual Differences, 20*, 402-414.

Tijms, J. (2007). The development of reading accuracy and reading rate during treatment of dyslexia. *Educational Psychology, 27,* 273-294.

Tijms, J. (in revision). Effectiveness of a computerized treatment for dyslexia in a clinical care setting: Outcomes and moderators. *Educational Psychology*.

Tijms, J., Hoeks, J.J.W.M., Paulussen-Hoogeboom, M.C., & Smolenaars, A.J. (2003). Long-term effects of a psycholinguistic treatment for dyslexia. *Journal of Research in Reading, 26,* 121-140.

Torgesen, J. K. (2005). Recent discoveries on remedial interventions for children with dyslexia. In M. J. Snowling & C. Hulme (Eds.), *The science of reading: A handbook* (pp. 521-537). Oxford: Blackwell Publishing.

Raven, J., Raven, J. C., & Court, J. H. (1998). *Coloured progressive matrices*. Oxford, UK: Oxford Psychologists Press.

Vickers, A. J., & Altman, D. G. (2001). Analysing controlled trials with baseline and follow up measurements. *British Medical Journal, 323,* 1123-1124.

Appendix A: EEG experiments protocol

Preparation of materials

- Place the 6 external electrodes in appropriate order (ext5 & 6 : will be placed first as reference in mastoids. Ext.1 & 2 for horizontal eye movement left-right, and ext.3 & 4 for vertical eye movement down-up position respectively).

Put the stickers on these electrodes and fill them with electrode gel.

- Electrodes gel
- Two syringes filled with gel
- Pieces of sticky tape
- Measuring tape
- Tissues
- Scrub-crème
- Alcohol swabs
- Cotton swabs
- Cotton
- Velcro band (to fasten the cap)
- Caps (for 64 electrodes, small-yellow and small/medium-yellow/red sizes)

This preparation of material should be ready 15 minutes before the scheduled arrival of the participant.

Computers

In both computers the safe mode (3rd option "*Metingen*") should be selected in the initial screen when they are started. Otherwise antivirus and windows update might interfere in the analysis. The starting of the computers, preparation of files and connection check must be done before the participants come to the room. Time: ~5 minutes.

Biosemi computer

- Initiate the Biosemi Actiview software
- Load the config file "gorka.cfg" by clicking on *about Actiview/ load config file.*
- Always check that the Biosemi AD box is connected and recognized in the Biosemi computer (USB connection)
- Always check the battery indicator and replace battery if it is not 100% full (have always a battery charging in the lab)
- Keep in mind that by default the starting file, initiates the file with the "pause" button clicked so it will not start saving unless we click on "pause" again.

Presentation computer

- Insert instructions of what to do before the child enters the room.
- Prepare which experiment to load and the folders to load/save the files.
- Audio settings in Presentation (sound level, which audio card, hardware/software settings): Device: Primary Sound Driver. Sound format tested: 16-bit, 2 channels, 44100hz. Sound attenuation (0.0 – 1): 0.1. Volume level (0.0 – 1): 0.2 (control of general volume level). Sounds presented through headphones at about 65 db SPL, match by loudness with Praat software.
- Check all other settings (button devices, logfile naming etc)

Video computer

- Set instructions and starting point of video. We show the movie "Wall-E" in a laptop computer. The initial 5 minutes are not displayed to avoid the starting credits.

Time distribution

Introduction/preparation/instructions MMN................ *30 min.*

Baseline with eyes closed... *2* min.

Experiment MMN (1st part) .... 29 *min. + 5 min pause divided in:*

Block 1- AV200

Block 2 – AV

Pause

Block 3 - Auditory

Block 4 - AV

Pause .... *10 min.*

Instructions VWR .... 1 min.

Experiment VWR (complete) .... *12 min.*

Pause .... *5 min.*

Experiment MMN (2nd part) .... *36 min. block + 2x5 min. pauses:*

Block 5 - AV200

Block 6 - Auditory

Pause

Block 7 - AV

Block 8 – Auditory

Pause

Block 9 – AV200

Estimated total time = 2h 15 min

(MMN Block duration = 7.2 min)

Arrival to the lab

- Introduction text :

“Welcome. This is our EEG lab, we'll have the experiment here. It has two rooms, one in which you sit and one where we will be, not to disturb you during experiment. Let's wlk around so that you can see what we have here (showing computers, screens, caps and materials on the table and giving a short description).

The experiment is consisted of 3 parts. First you will do a task where you will hear two sounds "a" and "b." Than the second part will have to read some words and in the third part you will again listen to the sounds. When each of the experiments starts we'll give you more detailed instructions.

The whole experiment shouldn't last longer than 2 and half hours. We know that it is a bit long and that some parts are less interseting than others, but it would be of great help to us if you could do the whole experiment. But you have to remember one thing, if at any point you feel like you can't do it, you should tell us and we will stop. So remember, don't feel afraid of telling us to stop. It is important to us that you feel ok.

At the end of the experiment you will receive diploma and a present

So if you are ready, we can start. Maybe you would like to go to the restroom or to have some water or something else?

We will start with putting an special EEG cap and attaching some electrodes to it. We will explain everything while we are doing it to be faster. But if you don't understand something, ask and we'll try to explain it better.

Can you, please sit here?”

- Show and explain materials. Let them feel the plastic part of the syringe, the electrodes, etc.
- Give a written detailed description to tutor/parent (same as that sent in the informed consent letter).

Electrodes preparation steps

1. Measure head circumference + nasion-inion and choose a cap
2. Fasten the velcro band to the cap.
3. Prepare location for the external electrodes (also for frontal electrodes) : alcohol-scrub gel-water- dry.
4. Place the external electrodes in the mastoids, put extra gel.
5. Place the cap. Check that is well centred with the measurements of nasion-inion. Fast the Velcro band.
6. Fill the holes for the electrodes with gel.
7. Ask participants if he/she wants to go to the WC, etc.
8. Place electrodes in the cap.
9. Place external electrodes for eyes movements

10. Check on screen that the placement of electrodes is correct, the electrodes signal in the monopolar view and the electrodes offset. The offset of the electrodes should be between -20 mV and 20 mV, if this is not the case locate the electrodes out of this range and place them again, adding more gel and/or gently scrubbing the hair with the syringe.
11. Show the Biosemi screen to the participant and do some practice to see the most common artefacts (blinking, biting, yawning, movements...)

Instructions for the participant

Before a new task/experiment the following instructions are shown on screen. The assistants also explain them and make sure they are clear to the participant. They also inform about how many blocks, how long they last and when the next pause comes.

Tasks

MMN experiment:

Audiovisual “*Je ziet dadelijk steeds een letter op het scherm. Je hoort ook klanken. Luister en kijk goed naar de letters en klanken. Soms zie je een cadeautje op het scherm. Druk dan op de knop. Probeer zo stil mogelijk te zitten”.*

Auditory “*Je kijkt dadelijk naar een film zonder geluid. Je hoort ook klanken. Probeer zo stil mogelijk te zitten”*

Word Recognition experiment:

Words *“ Je ziet dadelijk steeds een woord op het scherm. Kijk goed naar de woorden. Soms wordt een woord herhaald, druk dan op de knop. Probeer zo stil mogelijk te zitten”.*

Symbols “*Je ziet dadelijk steeds een figuur op het scherm. Kijk goed naar de figuren (show symbols). Soms zie je dat een symbool herhaald. Druk dan op de knop. Probeer zo stil mogelijk te zitten”.*

Pauses

“ *Goed zo!!! even pauze” or “Prima!!even pauze!!!”*or “*Goed gedaan! Even pauze”* together with a cartoon of sponge bob.

End

“*Dit was de test, je hebt het heel goed gedaan” bedankt voor het meedoen!”* together with a cartoon of sponge bob.

Behavioural aspects

It is important to keep the participant motivated, relax and comfortable. The pauses should be taken as close to our schedule as possible. The assistants are important for all these aspect and must be actively interacting with the participants:

Arrival

Show the material and let them see/ interact with it (i.e. syringe). Explain in a clear way

what the material is for. Make the child feel comfortable in the lab and with the people in the lab.

Explain the general aspects of the experiment: approximate length, pauses, type of tasks, importance of sitting still, say that they can stop if feeling unease, etc.

Also let him know that there will some rewards when he/she finishes the experiment and also a diploma will be given to him to acknowledge his participation . Make the child feel safe and enthusiastic about the experiment.

During the preparation

While the assistant himself and the experimenter are placing the cap and electrodes. Describe the different steps, ask him/her to say if they feel uncomfortable (just once or twice, not too often) . If the participant find it boring or by any reason is taking longer than expected, talk to him or offer him a quick drink or snack. Make the child feel comfortable with the apparatus.

After the preparation the child can make a picture with the cap and the electrodes that will be used in the diploma.

During the experiments

Before each experiment, the instructions are displayed on screen and the assistant explains them to the participant making sure he/she understands them correctly. Also ask if he/she has any doubt about it.

During the pauses

At the end of each block give always positive feedback and only if needed remind the importance of sitting still in the next block. Make clear during the whole experiment when there will be pauses. Explain always the length of the pauses and what comes next. Have always a scheme of the experiment and show it so the participant knows at all time in which step he/she is.

In the short pauses he/she can adjust his/her position in the chair, do some stretching, have a drink or snack, do some drawing, and have a talk with the assistant and experimenter.

There will be also board games available. In the long pause the participant should also stand up take a walk through the labs, go to the toilet, play a board game with the assistant, etc.

End of the experiment

Give positive feedback, let him know that what his/her participation was very important and he/she completed all the task well. Give also and immediate reward.

Biosemi recording

When the participant sits prepared (all electrodes in place, connected and still) click on PLAY button (before this the Biosemi computer/software must be also ready, see above). Display the Biosemi software screen on the monitor visible to the participant (switch between monitors with the switch box).

General check of signal

In the monopolar view:

Are there any misplaced, thick, black or noisy lines even when the subject is still and

relax? If yes, check which electrodes are wrong and that the external electrodes are still on place (if these are the ones giving the distort signal).

Is the whole signal interrupted, distorted or noisy? If yes, check that the ground electrodes are well placed, connected or if there is enough gel in them.

Electrodes offset:

Keep the electrodes offset range between -20 mV and 20 mV. If an electrode is not in range, locate it and add more gel and /or gently scrub the hair with the syringe.

Artefacts check with the participant

- Show to the participant the Biosemi screen.
- Show and explain the different coloured lines and what happens when he moves
- Show blinking and later movement of eyes in the Bipolar display.
- In the Monopolar display explore other artefacts, such as head movements, biting, yawning or laughing.
- Explain them that the more relax and still they are the clearer and nice the lines will be:

"Als je gewoon rustig en ontspannen zit en het taakje uitvoert dan kunnen we hele mooie golfjes/golven meten"

Recording and pauses

Pauses between blocks

- The program shows the words " *Goed zo!!! even pauze" or "Prima!!even pauze!!!"*
- Pause the Biosemi software
- The assistant interacts with the participant (see 'behavioural aspects') – In case child sits still very well – *'heel goed gedaan, de golfjes zien er heel mooi uit wat kan je dat goed zeg!'* In case the child moves quite a lot '*goed gedaan, als je na de pauze nog wat rustiger blijft zitten kunnen ze nog mooier worden'*
- Once the participant is ready to continue, check that the signal is still ok and click again in the pause button of the Biosemi to continue recording (always make sure that the file size keeps increasing)
- Start the presentation file

Pauses within a block

If the participant moves a lot or by any other reason a pause is needed: press ESC in presentation, then the word ' pauze' should appear. To go further press the key set to continue.

Storage

- When starting a file keep in mind that by default the starting file, initiates the file with the " pause" button clicked so it will not start saving unless we click on "pause" again.
- Specify folder, subject number and experiment (e.j.D:/data/users/gorka/EEG_raw/ S01MMN)

Notes during recording

The experiments/assistants should write down any abnormality encountered during the recording, including any problem during the preparation, wrong signals or problems with the computer software. As well as behavioural aspects that can be consider relevant and could affect the recording and/or the motivation and comfort of the participant during the experiment.

Stimuli description

Visual stimuli

MMN experiment

- Letter " a" (500ms) presented in the centre of the screen in font white on black background. Printed in lower case font "Arial" at letter size 40.
- Colour picture of a drawing of a gift box presented to ensure that participants focus on the screen during the block.
- Between stimuli a white cross is presented in the centre of the screen.

VWR experiment

- 80 Dutch words are presented printed in lower case font "Arial" in white font on a black background, at letter size 40 bold. They are high frequency words known by the majority (90 %) of 6 year old children. Two conditions: 4 &5 letters and 6 & 7 letters words.
- Strings of symbols using a "3 elementSymbols-1600" font. Printed so they have the same size and characteristics as the words and the same two conditions.
- Between stimuli a white cross is presented in the centre of the screen.

Sound settings

MMN experiment

- Natural speech sounds /a/ (384 ms) and /o/ (348 ms) are presented. They are digitally recorded (sampling rate 44.1 Khz, 16 bit quantization) from a female speaker. They are presented through headphones at about 65 db SPL. They are match by loudness with Praat software.

VWR experimenter

No sounds are presented in this experiment.

Appendix B: Timeline trial

	Dec. 2011	Jan. 2012	Febr.	March	April	May	June	July
		Intervention at clinic: N = 25, 2 x per week						
Intervention group Dyslexia	Pretest Behavioral: N = 25						Posttest Behavioral: N = 25	
		ERP: N = 25					ERP: N = 25	
Control group Dyslexia	Pretest Behavioral: N = 25			ERP: N = 25			Posttest Behavioral: N = 25	
Chronological Age Control group	Pretest Behavioral: N = 25			ERP: N = 25			Posttest Behavioral: N = 25	
Tot. Behavioral Measurement	N = 75			N = 25			N = 75	
Total ERP measurement		N = 25		N = 50			N = 25	
